# A compartmental model for smoking dynamics in Italy: a pipeline for inference, validation, and forecasting under hypothetical scenarios

**DOI:** 10.1186/s12874-024-02271-w

**Published:** 2024-07-13

**Authors:** Alessio Lachi, Cecilia Viscardi, Giulia Cereda, Giulia Carreras, Michela Baccini

**Affiliations:** 1 Department of Statistics, Computer Science, Applications “Giuseppe Parenti” (DiSIA), University of Florence, Viale Giovanni Battista Morgagni 59/65, Florence, 50134 Italy; 2 Epidemiology and Health Research Lab, Institute of Clinical Physiology of the Italian National Research Council (IFC-CNR), Via Giuseppe Moruzzi 1, Pisa, 56124 Italy; 3 Florence Center for Data Science, University of Florence, Viale Giovanni Battista Morgagni 59, Florence, 50134 Italy; 4 Oncologic Network, Prevention and Research Institute (ISPRO), Servizio Sanitario della Toscana, Via Cosimo il Vecchio 2, Florence, 50139 Italy

**Keywords:** Compartmental models, Smoking dynamics, Tobacco control policies, Global sensitivity analysis, Parametric bootstrap, Cross validation, Smoking attributable deaths, Forecasting, Calibration, Regression splines

## Abstract

We propose a compartmental model for investigating smoking dynamics in an Italian region (Tuscany). Calibrating the model on local data from 1993 to 2019, we estimate the probabilities of starting and quitting smoking and the probability of smoking relapse. Then, we forecast the evolution of smoking prevalence until 2043 and assess the impact on mortality in terms of attributable deaths. We introduce elements of novelty with respect to previous studies in this field, including a formal definition of the equations governing the model dynamics and a flexible modelling of smoking probabilities based on cubic regression splines. We estimate model parameters by defining a two-step procedure and quantify the sampling variability via a parametric bootstrap. We propose the implementation of cross-validation on a rolling basis and variance-based Global Sensitivity Analysis to check the robustness of the results and support our findings. Our results suggest a decrease in smoking prevalence among males and stability among females, over the next two decades. We estimate that, in 2023, 18% of deaths among males and 8% among females are due to smoking. We test the use of the model in assessing the impact on smoking prevalence and mortality of different tobacco control policies, including the tobacco-free generation ban recently introduced in New Zealand.

**Supplementary Information:**

The online version contains supplementary material available at 10.1186/s12874-024-02271-w.

## Background

Smoking is a significant risk factor for many common chronic diseases, including cancer, cardiovascular, cerebrovascular and respiratory diseases, diabetes, and a leading preventable cause of premature death [1, 2]. Also, smoking reduces length and quality of life [3], and contributes to health inequities [4]. The Global Burden of Disease study [5] reports that in 2019 smoking was responsible for around 8,709,000 deaths in the World (15.4% of all deaths), 907,000 in Europe, and 96,000 in Italy.

The importance of Tobacco Control Policies (TCP) has been firmly established within the World Health Organization’s (WHO) Framework Convention on Tobacco Control (FCTC), an international treaty that came into force in 2005 and has been ratified by 182 countries. Specifically, tobacco control has been included as one of the global development goals, recognized as crucial and necessary to achieve a one-third reduction in premature mortality by 2030 [6].

Focusing on Italy, data from the Italian surveillance system PASSI (Progressi delle Aziende Sanitarie per la Salute in Italia) highlighted that in 2021 23.7% of Italians (27.2% in men and 20.2% in women) described themselves as current smokers [7]. Among adolescents, smoking prevalence stalled in the last years, with a prevalence of current smokers between 27.3% and 32.4% in young people aged 13-16 years [8, 9].

Dynamic simulation models are widely used to describe and project the evolution of smoking habits in the population over time and to estimate the impact of past and hypothetical future TCPs. Since the 2000s, several models have been proposed [10–13], some of which developed within the Cancer Intervention and Surveillance Modelling Network (CISNET), a consortium of investigators funded by the National Cancer Institute, that uses mathematical modelling to study the impact of cancer control interventions [14]. These models are mainly of two types: compartmental models and agent-based models. Compartmental models, starting from a baseline year, perform macro-simulations so that the population evolves through deaths, births and changes in smoking habits [10–12]. The SimSmoke model [15] is the most used compartmental model [16], implemented for a wide number of countries including Italy [17–20]. Agent-based models, also called micro-simulation models, simulate individual life trajectories and interactions with a view to assessing their effects on the system as a whole [21, 22].

In this paper, grounding on previous works [12, 23–25], we developed a compartmental model that describes the evolution of smoking habits in Tuscany, a region of Central Italy, from 1993 to 2019, and forecasts them until 2043. The model assumes that at each point in time, the population is divided into non-overlapping groups called compartments, defined according to smoking status (never, current, and former smokers), age and sex [26]. Transitions between compartments are described by simple probabilistic rules and the evolution of the size of the compartments is governed by a system of differential equations.

While some of the transition parameters in the model were assumed as fixed, we estimated via a two-step calibration the age-specific probabilities of starting and quitting smoking, modelled in a flexible way through cubic regression splines [27], the probability of relapsing smoking, modelled as a nonlinear function of the time from quitting [28], and the mortality rate. We calibrated the model on the observed prevalence of never, current, and former smokers for the years from 1993 to 2019, arising from yearly local surveys.

Once we estimated the transition parameters, we predicted the prevalence of never, current, and former smokers in the regional population over time, and quantified the impact of smoking in terms of the number of smoking-attributable deaths (SAD) and population attributable fraction (PAF). With simple examples, we also illustrated the use of the compartmental model to predict the future impact of hypothetical interventions that act on the probabilities of starting and quitting smoking.

Compared to previous studies that dealt with the same problem, we aimed at presenting some methodological advances both in the modelling and estimation strategies. First of all, grounding on a formal definition of the model equations, we addressed the problem of accounting for sampling variability and provided confidence intervals for the estimates of the parameters and compartment sizes. To this end, due to the unavailability of the likelihood function associated with the model, we relied on a parametric bootstrap procedure [29, 30]. Also, we introduced a flexible modelling of the probabilities of starting and stopping smoking, usually assumed as constant, allowing them to change over time as functions of age. Moreover, we assessed the predictive performance of the model using cross-validation on a rolling basis. Finally, we assessed parameter identifiability through Global Sensitivity Analysis (GSA) [31].

## Methods

### Data

The analyses relied on data from heterogeneous sources. We used data from the National Institute of Statistics (ISTAT) Multipurpose Surveys “Aspect of Daily Life" (AVQ) (www.istat.it/it/archivio/91926), which every year collect fundamental information related to the daily life of individuals and families in Italy, enrolling about 25,000 families distributed in about 800 Italian municipalities of different population sizes. Specifically, we obtained from the ISTAT AVQ surveys an estimate of the distribution by smoking habit (never, current, and former smokers) of the population residing in Tuscany for each year from 1993 to 2019, separately for males and females and by age class (14-17, 18-19, 20-24, 25-34, 35-44, 45-54, 55-59, 60-64, 65-74, 75+). We obtained from the same surveys the smoking intensity distribution for current smokers, by sex and age class.

We used data from the ISTAT Multipurpose Surveys European Health Survey (EHIS) (www.istat.it/it/archivio/167485), a survey on the main aspects of public health carried out every 5 years from 1980 in all member states of the European Union, to obtain an estimate of the smoking intensity distribution among former smokers, as well as information about time since smoking cessation, separately for males and females and by the same age classes reported above. In particular, we considered the surveys for 1994, 1999, 2004, and 2013.

We obtained the size of the Tuscany population on January 1st 1993 and January 1st 2005, by age and sex, from the ISTAT website (www.istat.it). From the same website, we got the mortality rates by age and sex and the number of new births in Tuscany for the period 1993-2019. The relative risks (RRs) of death for smokers and ex-smokers versus never smokers are those reported in the Appendix of [32].

### Model specification

We specified a compartmental model for smoking habit dynamics in the population, which we call Smoking Habits Compartmental (SHC) model. In order to better present the SHC model adopted for the analysis, we first introduce a simpler version of it, and then proceed step by step, adding elements of complexity.

**Fig. 1:**
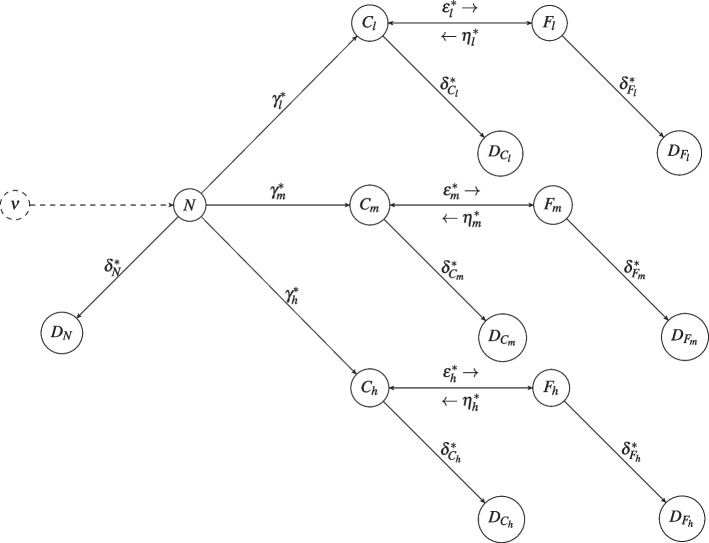
Smoking Habits Compartmental model in its simplest form

The starting model assumes that at each time the alive population is divided into the following non-overlapping compartments: never (*N*), current (*C*), and former (*F*) smokers. We consider only cigarette smoking. Never smokers can become current smokers, current smokers can become former smokers, and former smokers may restart smoking (smoking relapse). The compartments *C* and *F* are further divided into sub-compartments denoted by $C_i$ and $F_i$, where $i\in \{l,m,h\}$ indicates the level of smoking intensity, corresponding to low (<10 cigarettes/day), medium ($\ge$10 and <20 cigarettes/day), and high ($\ge$20 cigarettes/day) smoking intensity, respectively. During their life, individuals can change their smoking status, but, for the sake of simplicity, we assume that they cannot change their level of smoking intensity. The model admits deaths and new births. From each compartment, subjects can transit to a deceased compartment denoted by the letter *D* and a subscript corresponding to the compartment of origin. New births ($\nu (t)$ is the number of new births at time *t*) increase the size of the compartment *N*. Transitions of the individuals from a given compartment to another one determine flows regulated by the transition parameters, among which the rates of starting smoking ($\gamma _i^*$), stopping smoking ($\epsilon _i^*$), and relapsing into smoking after having stopped ($\eta _i^*$). Note that these rates can depend on the level of smoking intensity *i*. Death happens with different rates for never ($\delta ^*_N$), current ($\delta ^*_{C_i}$), and former ($\delta ^*_{F_i}$) smokers. For current and former smokers, the mortality rates may depend also on smoking intensity. This compartmental model, graphically represented in Fig. 1, is expressed by the following system of differential equations for each $i\in \{l,m,h\}$:

1 $$\begin{aligned} \left\{ \begin{array}{l} \frac{dN(t)}{dt}=-N(t) \left( 1-\delta ^*_N\right) \gamma ^*-N(t)\delta ^*_N+\nu (t) \\ \frac{dC_i(t)}{dt}=-C_i(t) \left( 1-\delta ^*_{C_i}\right) \epsilon _i^*-C_i(t)\delta ^*_{C_i}+N(t)\left( 1-\delta ^*_N\right) \gamma ^*_i+F_i(t)\left( 1-\delta ^*_{F_i}\right) \eta _i^*\\ \frac{dF_i(t)}{dt}=-F_i(t)\left( 1-\delta ^*_{F_i}\right) \eta _i^*-F_i(t)\delta ^*_{F_i}+C_i(t)\left( 1-\delta ^*_{C_i}\right) \epsilon _i^*\\ \frac{dD_N(t)}{dt}=N(t)\delta ^*_N\\ \frac{dD_{C_i}(t)}{dt}=C_i(t)\delta ^*_{C_i}\\ \frac{dD_{F_i}(t)}{dt}=F_i(t)\delta ^*_{F_i}, \end{array}\right. \end{aligned}$$

where $\gamma ^*=\gamma ^*_l+\gamma ^*_m+\gamma ^*_h$ is the overall transition rate from the status of never smoker to the status of current smoker. The initial conditions of the system, i.e. the sizes of the compartments at time 0, set to the 1^st^ of January 1993, are $N(0)=n_0$, $D_N(0)=0$, $F_i(0)=f^i_0$, $C_i(0)=c^i_0$ and $D_{C_i}(0)=D_{F_i}(0)=0$ $\forall i \in \{l,m,h\}$, where $n_0$ is the number of never smokers in the considered population on the first day of the study period, and $f^i_0$ and $c^i_0$ are the number of ex-smokers and current smokers with smoking intensity *i*.

For computational reasons, it is convenient to discretise the system of differential equations in Eq. (1), assuming that the size of the compartments is constant during 1-year time steps. Hereafter, *t* will denote discrete time, with the year as a time-unit ($t\in \{1,...,T\}$), and we replace the system in Eq. (1) with a system of difference equations, where the annual probability of stopping smoking ($\epsilon _i$), and the annual probabilities of smoking relapse ($\eta _i$) are derived from the corresponding rates in Eq. (1), as well as the annual probabilities of death for never ($\delta _{N}$), current ($\delta _{C_i}$), and former ($\delta _{F_i}$) smokers. In particular, $\delta _N=1-\exp (-\delta ^*_{N})$, and $\epsilon _i=1-\exp (-\epsilon _i^*)$, $\eta _i=1-\exp (-\eta _i^*)$, $\delta _{C_i}=1-\exp (-\delta ^*_{C_i})$, $\delta _{F_i}=1-\exp (-\delta ^*_{F_i})$ with $i \in \{l,m,h\}$. Regarding the probabilities of starting smoking for never smokers, the overall annual probability $\gamma$ comes from the corresponding rate, $\gamma =1-\exp (-\gamma ^*)$, while $\gamma _i=\pi _{C_i}\gamma$, where $\varvec{\pi }=(\pi _{C_l}, \pi _{C_m}, \pi _{C_h})$ is the distribution of the level of smoking intensity among the new current smokers. Notice that, if $\lambda$ is the rate of occurrence of an event, the probability of experiencing at least one event in the time unit is $1-\exp (-\lambda )$. The resulting system of discretised equations for each $i\in \{l,m,h\}$ and $t\in \{1,..., T\}$ is:

2 $$\begin{aligned} \left\{ \begin{array}{l} N(t)=N(t-1)\left( 1-\delta _N\right) \left( 1-\gamma \right) +\nu (t)\\ C_i(t)=C_i(t-1)\left( 1-\delta _{C_i}\right) \left( 1-\epsilon _i\right) +N(t-1)\left( 1-\delta _N\right) \gamma _i+F_i(t-1)\left( 1-\delta _{F_i}\right) \eta _i\\ F_i(t)=F_i(t-1)\left( 1-\delta _{F_i}\right) \left( 1-\eta _i\right) +C_i(t-1)\left( 1-\delta _{C_i}\right) \epsilon _i\\ D_N(t)=D_N(t-1)+N(t-1)\delta _N\\ D_{C_i}(t)=D_{C_i}(t-1)+C_i(t-1)\delta _{C_i}\\ D_{F_i}(t)=D_{F_i}(t-1)+F_i(t-1)\delta _{F_i}, \end{array}\right. \end{aligned}$$

where $\nu (t)$ denotes the newborns in the year *t*. The initial conditions of the system coincide with those of the previous model in Eq. (1).

The SHC model extends the system in Eq. (2) to account for two additional discrete time axes: age and time since smoking cessation. The final model is a compartmental model with separate compartments for each discrete age (*a*), where also a stratification by years since smoking cessation (*c*) is introduced for former smokers. Two separate SHC models are specified by sex. The final SHC model is defined by the following system of equations for each $i\in \{l,m,h\}$ and $t\in \{1,..., T\}$﻿:

3 $$\begin{aligned} \left\{ \begin{array}{ll} N(t;a)=\nu (t) &{} \text {if }a=0\\ N(t;a)=N(t-1;a-1)\left( 1-\delta _N(a-1)\right) \left( 1-\gamma (a-1)\right) &{} \text {if }a>0\\ C_i(t;a)=0 &{} \text {if }a=0\\ C_i(t;a)=C_i(t-1;a-1)\left( 1-\delta _{C_i}(a-1)\right) \left( 1-\epsilon (a-1)\right) + \\ \qquad \qquad N(t-1;a-1)\left( 1-\delta _N(a-1)\right) \pi _{C_i}\gamma (a-1) + &{} \\ \qquad \qquad \sum \limits _{c>0}F_i(t-1;a-1,c-1)\left( 1-\delta _{F_i}(a-1,c-1)\right) \eta (c-1) &{} \text {if }a>0\\ F_i(t;a,c)=0 &{} \text {if }a=0,\;c\ge 0\\ F_i(t;a,c)=C_i(t-1;a-1)\left( 1-\delta _{C_i}(a-1)\right) \epsilon (a-1) &{} \text {if }a>0,\;c=0\\ F_i(t;a,c)=F_i(t-1;a-1,c-1)\left( 1-\delta _{F_i}(a-1,c-1)\right) \left( 1-\eta (c-1)\right) &{} \text {if }a>0,\;c>0\\ D_N(t;a)=0 &{} \text {if }a=0\\ D_N(t;a)=D_N(t-1;a)+N(t-1;a-1)\delta _N(a-1) &{} \text {if }a>0\\ D_{C_i}(t;a)=0 &{} \text {if }a=0\\ D_{C_i}(t;a)=D_{C_i}(t-1;a)+C_i(t-1;a-1)\delta _{C_i}(a-1) &{} \text {if }a>0\\ D_{F_i}(t;a,c)=0 &{} \text {if }a=0,\;c\ge 0\\ D_{F_i}(t;a,c)=0 &{} \text {if }a>0,\;c=0\\ D_{F_i}(t;a,c)=D_{F_i}(t-1;a,c)+F_i(t-1;a-1,c-1)\delta _{F_i}(a-1,c-1) &{} \text {if }a>0,\;c>0. \end{array}\right. \end{aligned}$$

The initial conditions of the system are obtained by generalizing those of the model in Eq. (2), to take into account the stratification by age for current smokers, and the stratification by age and time since cessation for former smokers.

For simplicity, $\nu (t)$ was assumed to be constant over time. The age *a* takes values from 0 to 100. We set $\gamma (a)$ to 0 until 13 and from 35 years of age, and, in order to account for the possible non-linearity between 14 and 34, we modelled the *logit* transformation of $\gamma (a)$ through a natural cubic regression spline of age, with 2 equidistant internal knots. Similarly, we set $\epsilon (a)$ to 0 until 19 years of age; we introduced a natural cubic regression spline with 2 equidistant internal knots to model non-linearity for $a\ge 20$. The resulting functions are the following:

$$\begin{aligned} \gamma (a)= \left\{ \begin{array}{ll} 0 &{} \;0\le a\le 13\cup \;a\ge 35\\ \frac{\exp (s(a;\varvec{\psi }))}{1+\exp (s(a;\varvec{\psi }))}&{}\;14\le a\le 34 \end{array}\right. \qquad \epsilon (a)= \left\{ \begin{array}{ll} 0&{}\;0\le a\le 19\\ \frac{\exp (s(a;\varvec{\phi }))}{1+\exp (s(a;\varvec{\phi }))}&{}\;a\ge 20, \end{array}\right. \end{aligned}$$

where $\varvec{\psi }=(\psi _0,\psi _1,\psi _2,\psi _3)$ and $\varvec{\phi }=(\phi _0,\phi _1,\phi _2,\phi _3)$ are vectors of unknown parameters governing the probabilities of starting and quitting smoking, respectively. The relapsing rate, $\eta ^*(c)$, was modelled as a negative exponential function of the time since cessation, with parameters $\varvec{\omega }=(\omega _0,\omega _1$):

$$\begin{aligned} \eta ^*(c)= \left\{ \begin{array}{ll} 0&{}\;c=0\\ \omega _0\omega _1\exp (-\omega _1c)&{}\;1\le c\le 15\\ \omega _0\omega _1\exp (-\omega _115)&{}\;c\ge 16, \end{array}\right. \end{aligned}$$

where $\omega _0$ governs the lifetime probability of no relapse and $\omega _1$ tunes how fast the rate of smoking relapse declines with the time from cessation [12, 23, 28, 33]. Both $\omega _0$ and $\omega _1$ are assumed to be positive so that $\eta ^*(c)$ is a positive, decreasing function of *c*. The assumptions on which the SHC model is based are summarized in Section *Model assumptions*, Supplemental Material.

### Estimation strategy

An important issue in compartmental models concerns parameter identifiability [30]. Complex models with many compartments, such as the model in Eq. (3), have many parameters governing the admitted transitions, but unfortunately observed data are often insufficient to estimate all of them. To overcome this problem we fixed some of the parameters to values from the literature or external data, leaving as unknown the mortality risks and the spline coefficients $\varvec{\phi }$ and $\varvec{\psi }$, and $\varvec{\omega }$. Regarding the initial size of the compartments, it was obtained by combining the population size at the beginning of the study period with estimated prevalences arising from the ISTAT AVQ and EHIS surveys. Details on the values assigned to the fixed parameters and the initial size of the compartments are provided in Section S2, Supplemental Material. The unknown parameters have been estimated following the two step-procedure described in the next section.

#### Two-step estimation

In order to estimate the unknown parameters, we adopted a two-step procedure. Both steps use as observed data the prevalence of never, current and former smokers from ISTAT AVQ, here denoted by $p^{obs}(t;a^*)=\left( p^{obs}_C(t;a^*),p^{obs}_N(t;a^*),p^{obs}_F(t;a^*)\right)$, where *t* denotes the year and $a^*$ the age class. In particular, we considered years from 1993 to 2019 and age classes $a^*\in \{14-17, 18-19, 20-24, 25-34, 35-44, 45-54, 55-59, 60-64, 65-74, 75+\}$. According to the ISTAT AVQ survey, current smokers are defined as individuals who reported being smokers at the time of the interview, while former smokers as those who reported having quitted.

##### First step.

We estimated the age-specific risks of mortality for never smokers $\delta _N(a)$ using the prevalence values, as well as relative risks coming from the literature. In particular, the age-specific risks of dying for current and former smokers in the population at time *t* are respectively $\delta _C(t;a)=RR_{C}\times \delta _{N}(t;a)$ and $\delta _F(t;a)=RR_{F}\times \delta _{N}(t;a)$, with $RR_{C}$ and $RR_{F}$ the relative risks of dying for current smokers and former smokers versus never smokers. Let $p(t;a)=\left( p_N(t;a), p_C(t;a), p_F(t;a)\right)$ be the distribution of never, current and former smokers in the population. The overall mortality at age *a* in the year *t*, $\delta _{pop}(t;a)$, is a weighted average of $\delta _N(t;a)$, $\delta _C(t;a)$, and $\delta _F(t;a)$ with weights *p*(*t*; *a*). Thus, $\delta _N(t;a)$ can be derived as the ratio:

4 $$\begin{aligned} \delta _N(t;a)=\frac{\delta _{pop}(t;a)}{p_N(t;a)+RR_Cp_C(t;a)+RR_Fp_F(t;a)}. \end{aligned}$$

Therefore, separately for each year *t* in the period 1993-2019, we obtained an estimate of $\delta _N(t;a)$, plugging into Eq. (4) the mortality risk at age *a* reported for Tuscany, the relative risks for current and former smokers versus never smokers [32], and the observed age-specific prevalence of never, current and former smokers $p^{obs}(t;a^*)$. Finally, we averaged the year-specific $\hat{\delta }_N(t;a)$ over *t*, obtaining the overall estimate $\hat{\delta }_N(a)$. The risks of dying for current and former smokers by *i* and *c* were then derived as:

$$\begin{aligned} \hat{\delta }_{C_{i}}(a)=RR_{C_i}\times \hat{\delta }_{N}(a) \qquad \hat{\delta }_{F_i}(a,c)=RR_{F_i}(c)\times \hat{\delta }_{N}(a). \end{aligned}$$

##### Second step.

After fixing the mortality risks to the values computed at the first step, $\hat{\varvec{\delta }}(a,c)$, we calibrated the model on the observed prevalence $p^{obs}(t;a^*)$ to estimate the vector of parameters which were still unknown, $\varvec{\theta }=(\varvec{\psi },\varvec{\phi },\varvec{\omega })$. Let $p(t;a^*,\varvec{\theta })=\left( p_{C}(t;a^*,\varvec{\theta }),p_{N}(t;a^*,\varvec{\theta }),p_{F}(t;a^*,\varvec{\theta })\right)$ be the vector of the prevalence of never, current and former smokers belonging to the class of age $a^*$ at time *t*, calculated on the population predicted by the model in Eq. (3), given a specific value of $\varvec{\theta }$. With calibration, we searched for the value of $\varvec{\theta }$ that leads to predicted prevalences as close as possible to the observed ones. To compare observed and simulated trajectories, we considered the following objective function, where $H(\cdot ,\cdot )$ denotes the Hellinger distance [34] between two discrete probability distributions:

5 $$\begin{aligned} Obj(\varvec{\theta }){} & {} =\dfrac{1}{T\times A^*} \sum \limits _{t,a^*} H \left( p(t;a^*,\varvec{\theta }),p^{obs}(t;a^*) \right) \nonumber \\{} & {} = \dfrac{1}{T\times A^*\times \sqrt{2}} \sum \limits _{t,a^*} \sqrt{\sum \limits _{k\in \{C,N,F\}} \left( \sqrt{p_k(t;a^*,\varvec{\theta })}-\sqrt{p^{obs}_k(t;a^*)} \right) ^2}, \end{aligned}$$

where $A^*$ is the number of age classes $a^*$. We minimized the objective function in Eq. (5) over $\varvec{\theta }$ via a global optimization procedure, resorting to the JULIA package Optim.jl [35]. It is well-known that, in the context of compartmental models, optimization results often depend on the chosen starting points of the algorithm [36, 37]. To avoid the problem of getting stuck in local minima, we performed several optimizations using different starting points, then we selected the solution that brought to the minimum Hellinger distance [30, 36]. The two-step procedure was performed separately by sex, obtaining different estimates for males and females and sex-specific evolution of the compartment sizes. We estimated the compartment sizes up to 2043 by projecting the model dynamics, assuming that parameters and model structure do not change after 2019.

#### Parametric bootstrap procedure

We quantified the sampling variability around point estimates and projections by using a parametric bootstrap procedure [29, 30]. Let $\hat{\varvec{\theta }}$ be the vector of parameters minimizing the objective function in Eq. (5) and $p(t;a^*,\hat{\varvec{\theta }})$ the corresponding estimated vector of prevalence for never, current and former smokers of age $a^*$ in the population at time *t*. Let $n(t;a^*)$ be the number of subjects belonging to the age class $a^*$, enrolled in the ISTAT AVQ in the year *t* in Tuscany (i.e. the denominator of the observed prevalence $p^{obs}(t;a^*)$). The bootstrap procedure consisted of the following steps:

1. for each $a^*$ and *t*, we sampled a vector of prevalence from a Dirichlet distribution:
  6 $$\begin{aligned} p^b(t;a^*)\sim Dirichlet \left( p_C(t;a^*,\hat{\varvec{\theta }})n(t;a^*),p_N(t;a^*,\hat{\varvec{\theta }})n(t;a^*),p_F(t;a^*,\hat{\varvec{\theta }})n(t;a^*)\right) ; \end{aligned}$$
2. we considered the collection of these sampled vectors as the observed values and performed the two-step estimation, computing the vector $\varvec{\delta }^b(a,c)$ and finding $\varvec{\theta }^b$ that minimized the objective function;
3. we repeated the previous two steps $B=1000$ times, collecting a sample of *B* bootstrap estimates of $\varvec{\delta }(a,c)$ and $\varvec{\theta }$ to be used to estimate as many curves describing the transition parameters and compartment size trajectories;
4. we calculated the $90\%$ confidence intervals for the quantities of interest as the 5^th^ and 95^th^ percentiles of the bootstrap estimates; pointwise confidence intervals were calculated for the curves.

### Model validation

In order to evaluate the predictive performance of the estimation procedure described in Estimation strategy section, we applied cross-validation (CV) on a rolling basis. We started defining the first 3 years of the period 1993-2019 as the training set, and the subsequent *q* years as the test set. Then, we calibrated the compartmental model in Eq. (3) on the training set and used the estimated model to forecast the prevalence of never, current and former smokers in the years belonging to the test time window. The discrepancy between observed and projected prevalence was evaluated in terms of absolute percentage error. Then, we progressively extended the length of the training set by adding one year at a time, and we obtained the projections for the *q* subsequent years every time. We stopped when the last training set considered the years between 1993 to 2019-*q*. We finally computed the Mean Absolute Percentage Error (MAPE), by averaging the absolute percentage errors across different types of smokers over time, age classes, and training sets. Note that in general, for a set of *n* observations, MAPE is defined as $\frac{100}{n}\sum \nolimits _{i=1}^{n}{\frac{|O_i-E_i|}{O_i}}$, where $O_i$ is the observed value and $E_i$ is the expected one for unit *i*. We calculated the MAPE for different forecasting horizons by setting $q=3,6,9,12$ years.

### Sensitivity analysis

A key assumption of our model is that the dynamics of the studied phenomenon, particularly the transition probabilities between compartments, remain constant from 1993 to 2019 (and continue to do so until 2043). To verify its appropriateness, we conducted two separate analyses, first calibrating the model on the period 1993-2004 and then on the period 2005-2019, and compared the results. Notice that in the analysis 2005-2019 the initial sizes of the compartments were set to values obtained from 2005 surveys (see Section *Details on the fixed parameters*, Supplemental Material).

Another crucial point concerns the fact that the inference results could be affected by the model parameters assumed as fixed. To address this issue, we utilized a variance-based approach to Global Sensitivity Analysis (GSA) [31]. Given $K_X$ mutually independent inputs $(X_1, X_2,..., X_{K_X})$ and a model which, given the inputs, returns $K_Y$ outputs $(Y_1, Y_2,..., Y_{K_Y})$, this approach quantifies the relative importance of each input to the model’s outcomes by propagating uncertainty from the inputs to the outputs and computing variance indices. In our application, given the model in Eq. (3), we considered as inputs all the parameters, both fixed and unknown, and the Hellinger distance in Eq. (5) as the output *Y*. Note that, considering the Hellinger distance as the output, we directly measure the influence of the inputs on the discrepancy between observed and predicted data, thus, ultimately, on the inference results. Then we calculated, for each input $X_i$, the so-called total variance index, which ﻿$S^{tot}_i$ measures the overall effect of the *i*-th input on the output *Y*, including all the interactions of $X_i$ with the other inputs. This index corresponds to the expected variance of *Y* that would be left on average when all the parameters but $X_i$, $X_{\sim i}$, are fixed:

$$\begin{aligned} S^{tot}_i=\frac{E_{X\sim i}(Var_{X_i}(Y|X_{\sim i}))}{Var(Y)}. \end{aligned}$$

A total variance index close to zero indicates that the parameter $X_i$ does not influence *Y*, and therefore, the inference results. Conversely, a large total variance index indicates that the parameter does have an impact on them. In the former case, the parameter can be fixed without affecting our estimates, or in other words, our model and data do not provide information on this parameter. The computation of $S^{tot}_i$ relies on Monte Carlo simulations [38]. We simulated $K=10,000$ different combinations of the model inputs, then, for each of them, we predicted the prevalence values via the model in Eq. (3) and calculated the corresponding Hellinger distance. Specifically, we draw the model parameters from the distributions reported in Table S3.1, Supplemental Material, adopting a quasi-random numbers sampling which provides a more efficient exploration of the sample space [39, 40]. On the basis of the simulated Hellinger distance and the combination of the parameters, we computed the total variance indices as described in [38]. It is worth noting that in the GSA we did not include the age-specific mortality rates, $\delta _{pop}(t;a)$, among the model inputs. It is reasonable, as done elsewhere [23], to treat these parameters as not affected by uncertainty, given that they were estimated based on the entire population.

### Health impact assessment

The impact of smoking on population health was quantified in terms of attributable deaths. We calculated the Smoking-Attributable Deaths (SADs) in the year *t* as the difference between the number of deaths occurring in that year under the actual scenario, i.e. the number of deaths predicted by the model in Eq. (3) given $\hat{\varvec{\theta }}$ and $\hat{\varvec{\delta }}(a)$, and the deaths we would observe under a specific counterfactual condition. We considered the counterfactual condition where current smokers and former smokers in the year *t* were never smokers. Therefore, for each age *a*, we applied to the size of the compartments of smokers or ex-smokers the excess risk relative to never-smokers. The excess risk is defined as the difference between risks. For example, the excess risk of current smokers of age *a* and smoking intensity *i* relative to never-smokers is $\delta _{C_i}(a,c)-\delta _N(a)$. Thus, for the year *t*, the number of SADs among people of age *a* was calculated as:

$$\begin{aligned} \text {SAD}(t;a)=\sum \limits _iC_i(t,a; \hat{\varvec{\theta }})(\hat{\delta }_{C_i}(a)-\hat{\delta }_N(a))+ \sum \limits _i\sum \limits _cF_i(t,a,c; \hat{\varvec{\theta }})(\hat{\delta }_{F_i}(a,c)-\hat{\delta }_N(a)). \end{aligned}$$

The age-specific $\text {SAD}(t;a)$ can be summed over *a* to obtain the total number of attributable deaths in population or in a certain class of age: $\text {SAD}(t)=\sum \limits _a\text {SAD}(t;a)$. The impact of smoking on population health can be expressed also in terms of Population Attributable Fraction (PAF), defined as the proportion of deaths that would be avoided if all current and former smokers in the population or in a subset of it were never smokers [41]. For details, see Section *Population Attributable Fraction computation*, Supplemental Material. We calculated SADs and PAFs over the period 1993-2043, separately by sex and for the ages 35+ and 65+.

#### Impact of future hypothetical policies

In order to illustrate the use of the compartmental model to assess the impact of hypothetical TCPs on SAD, we focused on three policies acting on the rates of starting and stopping smoking, $\gamma ^*(a)$ and $\epsilon ^*(a)$. We assumed that all the defined policies are implemented in 2023 and that, in the absence of policies, the smoking habit dynamics would not change.

Taking inspiration from [42], and from a recent policy introduced in New Zealand (www.bbc.com/news/world-asia-63954862) we defined the following hypothetical TCPs starting from 2023:

- TCP1, a policy able to reduce the rate of starting smoking by 25% in 10 years for subjects between 14 and 34 years of age; for simplicity, we assumed a linear decrease, starting with a decrease of 2.5% the first year, a decrease of 5% the second one and so on, up to a final decrease of 25% after 10 years;
- TCP2, a policy able to increase the rate of stopping smoking by 25% in 10 years for subjects between 25 and 100 years of age; for simplicity, we assumed a linear growth, starting with an increase of 2.5% the first year, an increase of 5% the second one and so on, up to a final increase of 25% after 10 years;
- TCP3, a policy that imposes a complete smoking ban on cohorts born since 2009.

For each policy, we calculated the evolution of smoking prevalence and the number of avoided deaths expected from its implementation, taking the scenario without policies as a reference (TCP0). To better appreciate the impact of policies in terms of SAD, limited to this analysis, we extended the projections up to 2063.

## Results

The Tuscany population in 1993 counted 1,697,495 million males and 1,824,090 million females, and the proportions of never, current, and former smokers estimated from the ISTAT AVQ survey were respectively 35%, 34%, 31% for males and 67%, 20%, 13% for females.

**Fig. 2:**
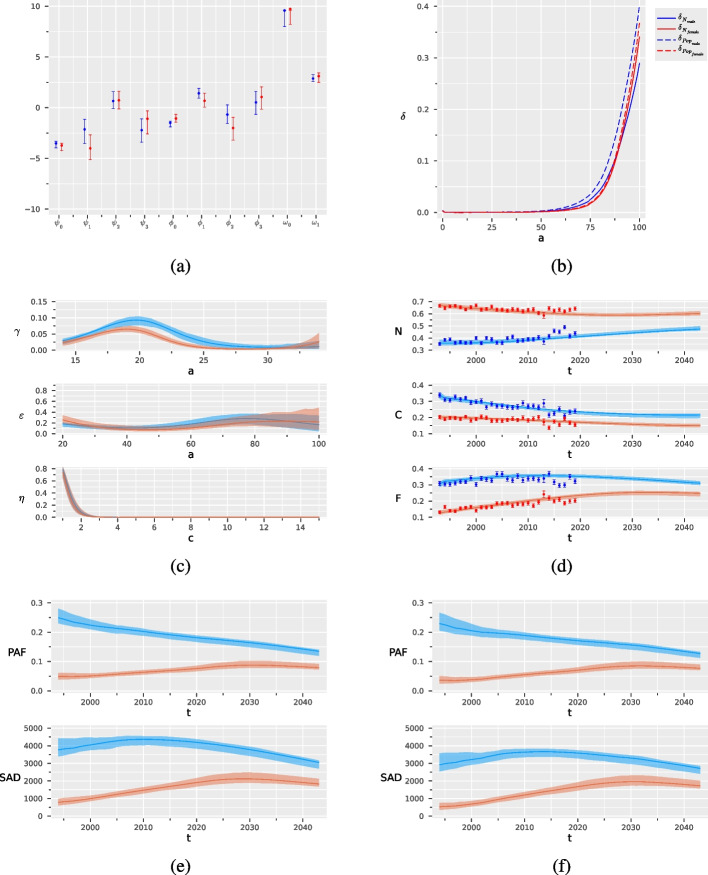
Results of the two-step estimation procedure for males in blue and females in red, with their bootstrap $90\%$ confidence intervals: parameters tuning the probabilities of starting ($\varvec{\psi }$) and stopping smoking ($\varvec{\phi }$), and the probability of smoking relapse ($\varvec{\omega }$) (**a**), age-specific mortality for never smokers and for the general population (**b**), probabilities of starting ($\gamma (a)$), and stopping smoking ( $\epsilon (a)$) and probability of smoking relapse ($\eta (c)$) (**c**), observed and predicted prevalence for never (*N*), current (*C*) and former (*F*) smokers (**d**), Population Attributable Fraction (PAF) and Smoking Attributable Deaths (SAD) for people over the age of 35 (**e**) and 65 (**f**)

Figure 2, Panel (a) and (b) show, separately for males and females, the estimates of the parameters left unknown in the SHC model in Eq. (3), with their 90$\%$ confidence intervals (CI), as obtained from the two-step estimation procedure and bootstrap. In particular, Panel (b) compares the estimated risk of death for never smokers with the one in the general population. It is worth noting that while the two risks are similar for females (the mortality among never-smokers is $8\%$ lower than among the general population), a not negligible difference is observed for males ($25\%$ lower) as noted also in [43].

Figure 2, Panel (c) shows the estimates of the probabilities of starting and quitting smoking and the probability of smoking relapse, derived from the estimated coefficients in Panel (a). Table 1 reports some summaries of the curves. Males are more likely to start and quit smoking than females. In particular, the probability of starting smoking has a peak around 19.9 years of age for males and 19.1 for females, with a maximum of just over 9% for males and just over 6% for females. The mean age of initiation is 20.7 for males and 20.5 for females. The probability of stopping smoking increases after 50 years of age, reaching a maximum of 29.5% for males and a maximum of 24.0% for females. The probability of smoking relapse is affected by large sampling variability. However, our results seem to indicate that it is about 80% after 1 year, then declines to about 40% after two years and progressively becomes negligible after 3 years (Fig. 2). On average, former smokers relapse into smoking after 1.7–1.5 years, for males and females respectively (Table 1).

**Table 1:** Summaries with 90% confidence intervals of the probabilities of starting and stopping smoking, and of the probability of smoking relapse, for males and females

Smoking event	Sex	Maximum probability	Age at maximum	**Mean probability**	Mean time at the event
**Starting smoking**	**Male**	0.09 (0.08 - 0.11)	19.9 (19.5 - 20.3)	0.04 (0.03 - 0.05)	20.7^a^ (19.9 - 21.6)
	**Female**	0.06 (0.05 - 0.08)	19.1 (18.6 - 19.9)	0.03 (0.02 - 0.04)	20.5^a^ (19.4 - 21.6)
**Stopping smoking**	**Male**	0.29 (0.19 - 0.62)	79.6 (70.3 - 100.0)	0.19 (0.10 - 0.30)	66.5^a^ (58.2 - 72.5)
	**Female**	0.24 (0.14 - 0.97)	20.1 (20.6 - 100.0)	0.10 (0.05 - 0.34)	57.2^a^ (52.2 - 74.9)
**Relapsing smoking**	**Male**	0.76 (0.63 - 0.79)	-	0.04 (0.03 - 0.05)	1.7^b^ (1.6 - 2.1)
	**Female**	0.69 (0.34 - 0.83)	-	0.02 (0.01 - 0.06)	1.5^b^ (1.4 - 2.3)

^a^Mean age of starting and stopping smoking

^b^Mean number of years from smoking cessation to relapse

Panel (d) shows the estimated prevalence of never, current, and former smokers among those over 14 years old from 1993 to 2043, predicted through the SHC model, together with the observed data used to calibrate the model (blue and red dots respectively for males and females with their 90% CI). The model fit appears to be adequate, with the predicted values close to the observed ones. Our forecasts, starting from 2020, suggest that the smoking prevalence will decrease in the coming years. Panels (e) and (f) show the predicted SAD and PAF over the period 1993-2043, separately for males and females, calculated for the population over 35 years of age and for the population over 65 years of age. The impact on males is higher than on females both in absolute and relative terms. However, while a clear reduction of the attributable deaths is expected in the coming years for males, for females they slightly decline only after having reached a maximum around 2030 [44]. Note that the majority of attributable deaths in the population over 35 are due to deaths in individuals over 65, as shown by the similarity of the curves.

Tables 2 and 3 report the percentages of never, current, and former smokers, the SAD and PAF, estimated every 10 years from 1993 to 2043, with their 90% confidence intervals. As an example, we estimated that in Tuscany in 2023 smoking was responsible for 4,070 (90% CI:3,795-4,247) deaths among men over 35 years old (one death per 745 people over the age of 35) and 1,976 (90% CI:1,741-2,407) deaths among women in the same age class (one death per 1655 people over the age of 35), corresponding to a PAF of 18% and 8%, respectively. Most of the attributable burden, however, was on people older than 65 (3,497 SAD for men and 1,765 for women).

**Table 2:** Estimated prevalence (%) of never, current, and former smokers in the population with 90% confidence intervals, evaluated every 10 years from 1993 to 2043, for males and females

	Never		Current		Former	
Year	Male	Female	Male	Female	Male	Female
**1993**	35.7 (33.6 - 37.4)	66.9 (65.2 - 68.8)	33.7 (31.9 - 35.4)	20.3 (19.0 - 21.8)	30.5 (28.3 - 33.1)	12.8 (11.4 - 14.1)
**2003**	36.5 (35.0 - 37.9)	63.4 (61.9 - 64.9)	28.7 (27.9 - 29.2)	19.4 (18.5 - 20.0)	34.8 (34.0 - 36.3)	17.2 (16.4 - 18.4)
**2013**	39.2 (38.1 - 40.6)	60.8 (59.5 - 62.0)	25.1 (24.1 - 25.5)	17.8 (17.2 - 18.6)	35.7 (34.8 - 37.0)	21.4 (20.4 - 22.2)
**2023**	42.2 (41.2 - 43.8)	59.1 (58.0 - 60.3)	22.9 (21.5 - 23.3)	16.5 (15.7 - 17.6)	34.9 (33.8 - 36.1)	24.5 (23.1 - 25.4)
**2033**	45.1 (44.0 - 47.1)	59.2 (58.0 - 60.8)	21.8 (20.0 - 22.3)	15.3 (14.5 - 16.7)	33.2 (32.0 - 34.3)	25.5 (23.7 - 26.4)
**2043**	47.4 (46.0 - 49.9)	60.3 (58.9 - 62.4)	21.6 (19.6 - 22.4)	14.8 (13.8 - 16.1)	31.0 (29.6 - 32.4)	25.0 (23.0 - 25.8)

**Table 3:** Estimated number of Smoking Attributable Deaths (SAD), Population Attributable Fraction (PAF) (%), and the ratio between overall age-specific population size and sex and age-specific SAD in the years 1993, 2003, 2013, 2023, 2033, 2043, 2053 and 2063, with 90% confidence intervals, among males and females aged over 35 and over 65

		SAD (90% CI)		PAF (90% CI)		Pop/SAD (90% CI)	
Age	Year	Male	Female	Male	Female	Male	Female
**35+**	**1993**	3,768 (3,390 - 4,273)	783 (625 - 965)	25.0 (23.0 - 27.4)	4.9 (3.9 - 6.0)	789 (695 - 877)	4,388 (3,558 - 5,499)
	**2003**	4,187 (3,948 - 4,403)	1,127 (985 - 1,312)	21.7 (20.4 - 22.8)	5.4 (4.7 - 6.2)	768 (728 - 816)	3,211 (2,756 - 3,674)
	**2013**	4,331 (4,076 - 4,498)	1,581 (1,402 - 1,877)	19.5 (18.4 - 20.2)	6.6 (5.9 - 7.8)	759 (730 - 808)	2,280 (1,916 - 2,574)
	**2023**	4,070 (3,795 - 4,247)	1,976 (1,741 - 2,407)	17.6 (16.4 - 18.3)	7.9 (7.0 - 9.6)	745 (713 - 801)	1,655 (1,353 - 1,884)
	**2033**	3,597 (3,316 - 3,749)	2,043 (1,873 - 2,496)	15.7 (14.5 - 16.4)	8.6 (7.8 - 10.4)	763 (731 - 831)	1,428 (1,164 - 1,564)
	**2043**	3,007 (2,746 - 3,153)	1,780 (1,628 - 2,341)	13.3 (12.1 - 14.0)	7.7 (7.0 - 10.1)	828 (788 - 910)	1,470 (1,111 - 1,610)
**65+**	**1993**	2,920 (2,555 - 3,445)	525 (369 - 704)	23.0 (20.7 - 26.0)	3.6 (2.5 - 4.8)	330 (280 - 378)	2,552 (1,903 - 3,635)
	**2003**	3,371 (3,128 - 3,592)	823 (672 - 1,017)	20.0 (18.6 - 21.2)	4.2 (3.4 - 5.1)	337 (315 - 366)	1,820 (1,470 - 2,232)
	**2013**	3,657 (3,423 - 3,811)	1,338 (1,164 - 1,589)	18.3 (17.1 - 19.1)	5.9 (5.2 - 7.0)	336 (322 - 360)	1,166 (977 - 1,341)
	**2023**	3,497 (3,239 - 3,663)	1,765 (1,532 - 2,165)	16.5 (15.5 - 17.4)	7.5 (6.5 - 9.1)	351 (334 - 381)	855 (692 - 991)
	**2033**	3,158 (2,887 - 3,319)	1,910 (1,743 - 2,356)	14.9 (13.7 - 15.7)	8.3 (7.6 - 10.2)	415 (394 - 458)	801 (644 - 881)
	**2043**	2,674 (2,439 - 2,829)	1,680 (1,541 - 2,233)	12.5 (11.4 - 13.3)	6.9 (6.9 - 9.9)	483 (455 - 533)	870 (647 - 951)

Regarding the CV procedure, the average values of MAPE for different prediction horizons are lower than 30% (Table 4), indicating that the predictive performance of the model is adequate, even if not optimal [45]. The MAPE is lower for the model on the male population than for the model on the female one.

**Table 4:** Cross-validation results: Mean Absolute Percentage Error (MAPE) calculated on four time horizons for the model on males and the model on females

Horizon	Male	Female
**3 years**	23.7	28.7
**6 years**	23.7	29.1
**9 years**	23.9	29.4
**12 years**	24.2	30.0

**Fig. 3:**
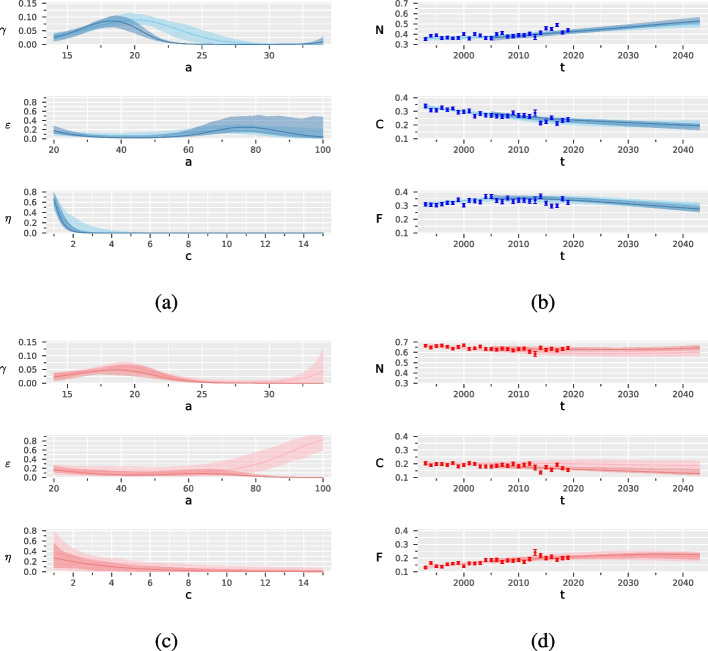
Results of the two-step estimation procedure for males in blue and females in red, by period of calibration (from 1993 to 2004 in a light colour and from 2005 to 2019 in a dark colour): probabilities of starting ($\gamma (a)$) and stopping smoking ($\epsilon (a)$), and probability of smoking relapse ($\eta (c)$), with 90% confidence bands, (**a**) and (**c**); prevalence of never (*N*), current (*C*) and former (*F*) smokers, with 90% confidence bands, (**b**) and (**d**)

Figure 3 reports the results of the two separate calibrations of the SHC model, one on the prevalence data from 1993 to 2004 and one on the prevalence data from 2005 to 2019. The confidence bands are wider in the second period of calibration than in the first one. For males, there is evidence of a downward shift of age corresponding to the maximum probability of starting smoking. For females, calibrating the model in the first years brought a lower projection of the prevalence of never smokers, which likely reflects a change over time in the smoking habits among women. Apart from these differences, the two calibrations provided qualitatively similar results. For numerical details see Tables S5.1-S5.6, Supplemental Material and Figures S5.1 and S5.2, Supplemental Material.

The total variance indices derived from the GSA (Table 5) reveal that the primary factor contributing to the variability of the Hellinger distance is the probability of starting smoking and its interaction with the other model inputs, resulting in $S_i^{tot}$ values of 0.58 for males and 0.76 for females. This is followed by the probability of quitting smoking, with values of 0.36 for males and 0.21 for females, and by the probability of smoking relapse, with values of 0.15 for males and 0.09 for females. Conversely, the parameters assumed to be fixed have a negligible impact on the Hellinger distance, with total variance indices very close to 0. This latter result indicates that fixing the aforementioned parameters to specific values does not significantly affect the calibration results, and consequently the prevalence estimates, demonstrating their robustness against variations in $\varvec{\pi }$, $\nu$, and RRs specifications.

**Table 5:** Total variance indices quantifying the contribution of each input on the Hellinger distance, calculated for the model on males and the model on females

Input	Male	Female
$\varvec{\psi }$	0.58	0.76
$\varvec{\phi }$	0.36	0.21
$\varvec{\omega }$	0.15	0.09
$\varvec{\pi }$	$<0.01$	$<0.01$
$\varvec{\nu }$	$<0.01$	$<0.01$
*RRs﻿*	$<0.01$	$<0.01$

**Fig. 4:**
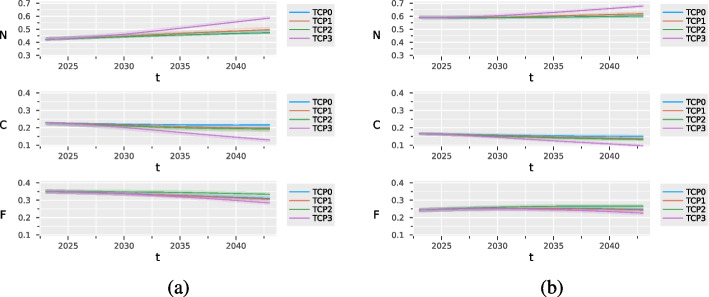
Estimated prevalence of never (*N*), current (*C*) and former (*F*) smokers under different tobacco control policies (TCP) with $90\%$ confidence bands, for males (**a**) and females (**b**)

Figure 4 compares the evolution of smoking habits in the male and female populations under three alternative scenarios that simulate hypothetical tobacco control policies. These scenarios are compared with the status quo, corresponding to the absence of actions to reduce tobacco consumption (TCP0). We assumed that the TCPs are applied since 2023. They have no substantial effect on the prevalence of never, former, and current smokers during the 10 years following their implementation. TCP3 has the largest impact: in 2043 it is expected to increase by 12 percentage points the prevalence of never-smokers among males and by 8 among females, compared with TCP0 (see Table 6).

**Table 6:** Estimated prevalence (%) of never, current, and former smokers in the population under different tobacco control policies (TCP) evaluated in 2023, 2033 and 2043, with 90% confidence intervals, for males and females

		Male				Female			
Status	Year	TCP0	TCP1	TCP2	TCP3	TCP0	TCP1	TCP2	TCP3
**Never**	**2023**	42.1 (41.2 - 43.8)	42.2 (41.2 - 43.8)	42.2 (41.2 - 43.8)	42.2 (41.2 - 43.8)	59.1 (58.0 - 60.3)	59.1 (58.0 - 60.3)	59.1 (58.0 - 60.3)	59.1 (58.0 - 60.3)
	**2033**	45.1 (44.0 - 47.1)	45.2 (44.9 - 48.0)	45.1 (44.0 - 47.1)	48.9 (47.8 - 50.8)	59.2 (58.0 - 60.8)	59.8 (58.6 - 61.4)	59.2 (58.0 - 60.8)	61.9 (60.6 - 63.4)
	**2043**	47.4 (46.0 - 49.9)	49.7 (48.3 - 52.0)	47.3 (45.9 - 49.8)	58.7 (57.4 - 60.5)	60.3 (58.9 - 62.4)	62.0 (60.7 - 64.0)	60.3 (58.9 - 62.4)	67.9 (66.5 - 69.5)
**Current**	**2023**	22.9 (21.5 - 23.3)	22.9 (21.5 - 23.3)	22.9 (21.4 - 23.3)	22.9 (21.5 - 23.3)	16.5 (15.7 - 17.6)	16.4 (15.7 - 17.6)	16.4 (15.6 - 17.5)	16.4 (15.7 - 17.6)
	**2033**	21.8 (20.0 - 22.3)	21.0 (19.2 - 21.5)	20.3 (18.6 - 20.8)	18.4 (16.8 - 19.0)	15.3 (14.5 - 16.7)	14.8 (13.9 - 16.1)	14.3 (13.4 - 15.3)	13.0 (12.2 - 14.4)
	**2043**	21.6 (19.6 - 22.4)	19.8 (17.9 - 20.5)	19.3 (17.4 - 20.0)	12.9 (11.4 - 13.5)	14.8 (13.8 - 16.1)	13.5 (12.7 - 14.8)	13.1 (12.2 - 14.4)	9.4 (8.6 - 10.7)
**Former**	**2023**	34.9 (33.8 - 36.1)	34.9 (33.8 - 36.1)	35.0 (33.9 - 36.2)	34.9 (33.8 - 36.1)	24.5 (23.1 - 25.4)	24.5 (23.1 - 25.4)	24.5 (23.1 - 25.5)	24.5 (23.1 - 25.4)
	**2033**	33.1 (32.0 - 34.3)	33.0 (31.9 - 34.1)	34.7 (33.4 - 35.8)	32.6 (31.5 - 33.8)	25.5 (23.7 - 26.3)	25.4 (23.6 - 26.2)	26.5 (24.5 - 27.4)	25.0 (23.3 - 25.9)
	**2043**	31.0 (29.6 - 32.4)	30.5 (29.0 - 31.9)	33.4 (31.8 - 34.8)	28.4 (27.0 - 29.8)	24.9 (22.9 - 25.9)	24.4 (22.4 - 25.3)	26.7 (24.6 - 27.6)	22.7 (20.6 - 23.4)

In order to better appreciate the impact of the TCPs on mortality, we extended the forecasting horizon up to 2063. Table 7 reports the predicted number of attributable deaths every 10 years, from 2023 to 2063, for both males and females under different TCPs, for the classes of age 35+ and 65+. TCP2, which increases the probability of stopping smoking, is the policy that most impact mortality in both classes of age. TCP3, which bans access to smoking to the new generations, despite its effectiveness in reducing current smokers, does not reduce SADs within the time window considered. Indeed, this policy is expected to have a longer-term impact, which is not visible before 2063. Additional Tables and Figures are reported in Section *Additional results*, Supplemental Material.

**Table 7:** Expected number of Smoking Attributable Deaths (SAD) under different tobacco control policies (TCP), in the years 2023, 2033, 2043, 2053 and 2063, with 90% confidence intervals, among males and females aged over 35 and over 65

		Male				Female			
Age	Year	TCP0	TCP1	TCP2	TCP3	TCP0	TCP1	TCP2	TCP3
**35+**	**2023**	4,070 (3,975 - 4,247)	4,070 (3,975 - 4,247)	4,070 (3,975 - 4,247)	4,070 (3,975 - 4,247)	1,976 (1,741 - 2,407)	1,976 (1,741 - 2,407)	1,976 (1,741 - 2,407)	1,976 (1,741 - 2,407)
	**2033**	3,597 (3,316 - 3,749)	3,597 (3,315 - 3,749)	3,547 (3,263 - 3,702)	3,597 (3,316 - 3,749)	2,043 (1,873 - 2,496)	2,043 (1,873 - 2,496)	2,010 (1,841 - 2,460)	2,043 (1,873 - 2,496)
	**2043**	3,007 (2,746 - 3,153)	3,005 (2,774 - 3,151)	2,843 (2,578 - 2,986)	3,007 (2,746 - 3,153)	1,780 (1,628 - 2,341)	1,779 (1,627 - 2,340)	1,672 (1,524 - 2,243)	1,780 (1,628 - 2,341)
	**2053**	2,401 (2,171 - 2,505)	2,389 (2,159 - 2,490)	2,182 (1,958 - 2,291)	2,352 (2,126 - 2,454)	1,432 (1,313 - 1,868)	1,427 (1,309 - 1,865)	1,295 (1,179 - 1,740)	1,418 (1,299 - 1,853)
	**2063**	1,889 (1,663 - 1,972)	1,855 (1,633 - 1,937)	1,664 (1,447 - 1,746)	1,739 (1,533 - 1,819)	982 (870 - 1,235)	969 (855 - 1,224)	855 (749 - 1,115)	936 (824 - 1,185)
**65+**	**2023**	3,497 (3,239 - 3,663)	3,497 (3,239 - 3,663)	3,497 (3,239 - 3,663)	3,497 (3,239 - 3,663)	1,765 (1,532 - 2,165)	1,765 (1,532 - 2,165)	1,765 (1,532 - 2,165)	1,765 (1,532 - 2,165)
	**2033**	3,158 (2,887 - 3,319)	3,158 (2,887 - 3,319)	3,114 (2,846 - 3,279)	3,158 (2,887 - 3,319)	1,910 (1,743 - 2,356)	1,910 (1,743 - 2,356)	1,879 (1,710 - 2,322)	1,910 (1,743 - 2,356)
	**2043**	2,674 (2,439 - 2,829)	2,674 (2,439 - 2,829)	2,529 (2,299 - 2,691)	2,674 (2,439 - 2,829)	1,680 (1,541 - 2,233)	1,680 (1,541 - 2,233)	1,578 (1,441 - 2,140)	1,680 (1,541 - 2,233)
	**2053**	2,073 (1,874 - 2,174)	2,073 (1,874 - 2,174)	1,885 (1,696 - 1,993)	2,073 (1,874 - 2,174)	1,334 (1,219 - 1,760)	1,334 (1,219 - 1,760)	1,207 (1,089 - 1,641)	1,334 (1,219 - 1,760)
	**2063**	1,550 (1,361 - 1,626)	1,548 (1,360 - 1,625)	1,368 (1,189 - 1,440)	1,550 (1,361 - 1,626)	881 (774 - 1,123)	879 (773 - 1,123)	768 (671 - 1,016)	881 (774 - 1,123)

## Discussion

Interesting findings emerged from our analysis. We found that the probability of starting smoking reaches its maximum, just over 9% for males and just over $6\%$ for females, between 19 and 20 years of age. Considering that younger people have a large probability of becoming stable smokers [46], these probabilities are quite worrying. The difference in the mean age of initiation between males and females is lower than one year, confirming what is reported for high-income countries [47]. Regarding the probability of stopping smoking, we found that it increases after 50 years of age and has a maximum of 29.5% for males and 24.0% for females, even if the confidence bands around these curves are quite wide. The 80% of ex-smokers relapse into smoking after 1 year, in line with the results of the Italian surveillance system PASSI for the years 2020-2021 (www.epicentro.iss.it/passi/dati/SmettereFumo). On average, former smokers relapse into smoking during the second year from cessation (after 1.7 and 1.5 years for males and females, respectively).

According to our model, in 2023 in Tuscany, 23% of men smoke, while 35% are ex-smokers. These percentages are lower among women: 16% smoke and 24% are ex-smokers. The prevalence of smokers estimated by our model is lower than the one reported in the PASSI survey for the period 2020-2021 (26.1% and 20.5% in the age class 18-69 for males and females, respectively), but consistent if we consider that our estimates are calculated on all population, while PASSI focuses on the age class 18-69 (www.epicentro.iss.it/passi/pdf2020/Scheda-fumo-PASSI-regione-2016-2019.pdf).

We estimated that, in 2023, 18% of deaths among males and 8% among females are due to smoking, corresponding to 4,070 and 1,976 deaths, respectively. These PAFs are in line with those estimated by the Global Burden of Disease Study for Italy in 2019 (https://vizhub.healthdata.org/gbd-results/), 20.5% (CI: 19.5-21.7) in males and 8.17% (CI: 7.51-9.02) in females, slightly lower than those reported for Italy by the Tobacco Atlas initiative (https://tobaccoatlas.org/challenges/deaths/), and overall coherent with previous results for Italy and Tuscany ([48]; www.deathsfromsmoking.net).

As shown by the cross-validation results, the model produces quite reliable predictions of prevalence. Thus, subject to the assumption that all mechanisms underlying smoking dynamics and demographic evolution do not change in the future, we projected the dynamics. For the next two decades, we estimated an evident decrease in the prevalence of current smokers for males, due to an increase in the percentage of never-smokers. For females, substantial stability is expected. Similar considerations apply to PAFs: a decrease is observed for males and stability for females. These results confirm that Italy is in the fourth stage of the tobacco epidemic model, characterized by a continuing slow decline of smoking prevalence in both men and women with converging rates between sex [49, 50]. The robustness of the long-term predictions to events that could change the described dynamics over time could be assessed by implementing a specific GSA procedure. However, in this work, we used the GSA only to assess the sensitivity of the inference results to changes in the parameters treated as fixed.

The proposed model can be used for assessing the impact of alternative TCPs. For illustrative purposes, we considered the impact of three policies aimed at reducing smoking in the population. The first two policies are completely hypothetical and defined in terms of their effect on the probability of starting (TCP1) and stopping (TCP2) smoking. They are not real policies but represent the intentions of the legislator to change the rates of smoking initiation and cessation. The third one (TCP3), which bans smoking in new cohorts since 2009, is inspired by the tobacco-free generation real intervention implemented in New Zealand as part of a plan for the tobacco endgame, including also additional strategies aimed at decreasing the affordability and availability of smoking, reducing the levels of nicotine in tobacco products, and restricting sales to designated tobacco outlets. We evaluated the expected marginal impact that this tobacco-free generation intervention would have in Tuscany, assuming complete compliance of new generations to the smoking ban. The results indicate that under TCP1 and TCP2 the prevalence of current smokers is reduced by a few percentage points either for women or men. On the contrary, TCP3 produces a clear increase in never-smokers, thus a reduction in smoking prevalence, which is expected to decrease in ten years by 9 and 6 percentage points among males and females, respectively. The impact on mortality of the three policies, in particular TCP1 and TCP3, that act by increasing the number of never smokers, can be appreciated only by extending the time horizon of forecasting. Interventions able to increase the probability of stopping smoking, like TCP2, are expected to produce the largest reduction of SADs in the medium term, especially among the over-65s. However, this kind of policy does not contribute to reducing smoking among the youngest, thus effectively stopping the tobacco epidemic.

From a methodological point of view, we introduced several elements of novelty. First of all, we provided a formal definition of the equations that describe the system dynamics and made explicit assumptions on the distribution of the involved random variables. We also introduced cubic regression splines for modelling in a flexible way the probabilities of starting and quitting smoking as functions of age, thus obtaining more realistic trajectories. Furthermore, we included in the model dependencies from the smoking intensity, which may allow assessing the impact of personalized TCPs specific for heavy or moderate smokers, such as lung cancer screening, use of pharmacological treatment, or smoking cessation campaigns.

Regarding the inference on the unknown parameters, we proposed a two-step estimation strategy to estimate the curves describing the probability of starting and stopping smoking and the probability of smoking relapse, as well as the mortality risk among never, current and former smokers. At the second step of the estimation procedure, we defined the calibration objective function in terms of a Hellinger distance between observed and predicted prevalence, instead of the widely used sum of squares function. The use of this discrepancy measure is relatively new in this framework and allowed handling a bounded loss function, defined in [0, 1], that is simple to minimise and to be interpreted. Finally, we provided confidence intervals/bands for the parameters/curves of interest. To the best of our knowledge, this is the first time that quantification of sampling variability is performed in this field. To this aim, we resorted to a parametric bootstrap procedure defined by adapting to our framework a method proposed for compartmental models describing infectious dynamics [30]. The assumed Dirichlet distribution enabled us to model the prevalence values complying with the constraint that their sum equals one. Furthermore, specifying appropriate values for the concentration parameters of the Dirichlet distributions, we were able to quantify the sampling variability accounting for the sample size of the surveys from which we derived the observed prevalence used in calibration. The estimation procedure has also limitations. We estimated the parameters in a deterministic way, in the sense that we considered the distributional assumptions on the prevalence only in the bootstrap procedure but not in the calibration phase. While likelihood-based approaches are unfeasible in this framework, likelihood-free inference methods such as Approximate Bayesian Computation algorithms would allow a full uncertainty quantification [25].

The reliability of the model’s results depends on several factors. First of all, it depends on the quality of the data used for calibration. In our case, we used data from yearly surveys conducted according to well-established methodology on reasonably large sample sizes. Secondly, it depends on the values of the fixed parameters, and we demonstrated, through the GSA, that the inference was robust to variations of the fixed parameters within plausible ranges of values. Lastly, the reliability of the results depends on the structural assumptions on which the model is based, not assessed via GSA. Underneath, we qualitatively review the main assumptions of the model and discuss the limitations that may arise from them. With respect to demographic dynamics, we assumed that the population was close to immigration and emigration and that the number of new births did not vary during the study period, effectively feeding the model with identical cohorts of subjects each year. For more realistic modelling, we could use the observed yearly number of births to create the new cohorts up to 2019. However, in light of the GSA, we expect that the impact of this choice on the results has not been significant. We also assumed that the age-specific mortality rates did not vary over the study period.

Regarding smoking dynamics, we assumed that the probabilities of starting and stopping smoking were functions of age and that the probability of smoking relapse was a function of time since cessation, but not of age. We did not allow any of these probabilities to vary over time. By defining the transition probabilities in this way, we have made a clear choice about which time axes were most important in our opinion to capture appropriately the smoking dynamics in the population. This choice is not without problems because in some cases there is evidence suggesting otherwise. For example, a decreasing trend in the probability of starting smoking has been reported for both males and females in Europe [51], while evidence of a dependence between age and risk of smoking relapse has been found in the US population [52]. However, if introducing multiple time-axes dependence in the transition probabilities could lead to more realistic results, this would be at the price of further complicating the model by introducing new unknown parameters to be estimated. We partially explored the goodness of the assumption of no calendar time dependence through a simple sensitivity analysis, which confirmed that the probabilities of starting and stopping smoking, and the probability of smoking relapse were quite similar when two separate calibrations were performed on the periods 1993-2004 and 2005-2019. It is worth stressing that, even if these two periods correspond to before and after the introduction of the so-called Sirchia law that banned smoking in all indoor public places in Italy, it was not our goal to speculate about the causal effect of this intervention on smoking dynamics. We also assumed that people could not change their smoke intensity during their entire life, that the probability of stopping and relapsing did not depend on smoking intensity, and, again, that the distribution of smokers by smoking intensity did not change over the study period.

In general, it is important to note that underlying all the simplifications introduced in model specification is the fact that adding details to a compartmental model goes along with the definition of new compartments and new transitions, and without available and reliable data, the model could become non-identifiable producing more uncertain and unstable results [53]. Moreover, microsimulation models or social network models, that explore smoking dynamics from an individual point of view, could be more suitable solutions to introduce detail and complexity, including those related, for example, to the course of disease [54, 55], or to explore the exposure to second-hand smoke that, being related to the social network of the individuals, was not considered in our analysis.

## Conclusions

We developed an approach for modelling smoking dynamics in the population that overcomes many of the limitations of previously proposed models. It includes validation tools like cross-validation on a rolling basis and GSA, aimed at checking the robustness of our results and supporting our findings.The model can be generalized and applied to other Italian regions changing the initial conditions of the system. The fact that the surveys we relied on provide information about all regions makes this extension easily feasible. The proposed approach can be straightforwardly applied also to other countries after a careful check of the validity of the model assumptions, which, however, can be mostly adapted to different contexts. Finally, it can be also used to assess the impact of other tobacco control policies on smoking prevalence and mortality, beyond those considered in this paper.

### Supplementary Information

Supplementary Material 1.

## Data Availability

Data are available on request from the corresponding author.

## Code availability

Codes are available on request from the corresponding author.
