# Supplementary material for: A compartmental model for smoking dynamics in Italy: a pipeline for inference, validation, and forecasting under hypothetical scenarios
Source: BMC Med Res Methodol. 2024 Jul 13;24:148. doi: 10.1186/s12874-024-02271-w (PMC11245805; doi:10.1186/s12874-024-02271-w)
Supplement: Supplementary file 1 — Supplementary Material 1. [file 12874_2024_2271_MOESM1_ESM.pdf]

Supplemental Material

S1 Model assumptions

The assumptions underlying the SHC model are summarized below:

- the probability of starting smoking $\gamma_i(a)$ depends on age and smoking intensity;
- people can start smoking between the ages of 14 and 34. Note that this assumption relaxes the one in Levy et al. [1], where the maximum initiation age was set to 24;
- the probabilities of starting smoking $\gamma_i(a)$ depend on the age through $\gamma(a)$, while the distribution of the level of smoking intensity $\boldsymbol{\pi}$ is assumed to be constant over time and age;
- the distribution of the new smokers by smoking intensity does not depend on age and calendar time;
- smokers do not change their smoking intensity during their entire life (this also implies that if an ex-smoker goes back to smoking, her/his smoking intensity is the same as when she/he first started smoking). This assumption is in line with evidence that smoking patterns tend to stabilize during the first years after smoking initiation [2]. It is also supported by evidence that the number of "reducers" and "increasers" is similar, leading to an overall balance of the transitions [3];
- the probability of stopping smoking $\varepsilon(a)$ depends only on age;
- people can quit smoking only after 20 years of age. Note that this assumption relaxes the one in Levy et al. [1], where smoking cessation is not allowed before age 25;
- the probability of relapsing $\eta(c)$ changes with time since smoking cessation, but does not depend on age and smoking intensity;
- after 15 years since smoking cessation, the probability of smoking relapse becomes constant;
- the rates of quitting depend on age but does not depend on the level of smoking intensity;
- an ex-smoker who first relapses, then stops smoking again, becomes a 0-year former smoker;
- the population is closed to immigration and emigration (but we considered new births and deaths);
- the risk of death depends on the age for never smokers ($\delta_N(a)$), both on smoking intensity and age for current smokers ($\delta_{C_i}(a)$), and on time since smoking cessation and age for former smokers ($\delta_F(a,c)$). For the reason of simplicity, we do not consider the level of smoking intensity in the definition of the mortality for former smokers: $\delta_{F_i}(a,c) = \delta_F(a,c)$ for each $i \in \{l,m,h\}$;
- the mortality rate of current smokers does not depend on the time from starting smoking;
- the mortality rate of former smokers does depend on the time from smoking cessation and on age;
- for each individual, only one event among starting, quitting, relapsing, being born or dying occurs in the year and we assume that it happens at the end of the year;
- at each time the probabilities of starting and quitting smoking and the probability of smoking relapse are defined among those who do not die during the year (they are conditional probabilities);
- all the transition rates do not change with t.

S2 Details on the fixed parameters

The values assigned to the fixed parameters of the SHC model are detailed below:

- the vector of proportions $\boldsymbol{\pi}$ was set to the average proportions of low, medium, and heavy smokers estimated for ages 34-44 from the ISTAT AVQ surveys (www.istat.it/it/archivio/91926) carried out from 1993 to 2019. Specifically, we set $(\pi_{C_l}, \pi_{C_m}, \pi_{C_h}) = (0.19, 0.40, 0.41)$ for males and $(\pi_{C_l}, \pi_{C_m}, \pi_{C_h}) = (0.36, 0.44, 0.20)$ for females (see Figure S2.1);
- $\nu(t)$ was assumed to be constant over time, being the new births quite stable over the years 1993-2019. In particular, we set $\nu(t)$ to the average number of new births in Tuscany from 1993 to 2019, $\nu(t) = 14,701$ for males and $\nu(t) = 13,895$ for females (http://www.istat.it/);
- the initial number of never, current and former smokers, by age and sex, was obtained applying to the resident population in Tuscany on the 1st of January 1993 ($t = 0$) (http://www.istat.it/) the prevalence of never, current, and former smokers estimated from the 1993 ISTAT AVQ survey (www.istat.it/it/archivio/91926), as well as the smoking intensity distribution from the ISTAT AVQ and EHIS surveys (www.istat.it/it/archivio/167485) for current and former smokers, respectively. For details on population size and the prevalence of never, current, and former smokers in 1993 see Figures S2.1-S2.3. Note that in the sensitivity analysis described in Section 3.4 of the manuscript, we used the same quantities referred to the year 2005 (Figures S2.4 and S2.5);
- in order to quantify $C_i(0;a)$, the initial number of current smokers in 1993, stratified by age, obtained as described at the previous point, has been multiplied by the proportions of low, medium and high-intensity smokers arising from the ISTAT AVQ survey carried out from 1993 to 2019 (Figure S2.1). This procedure has been applied separately for males and females;
- in order to quantify $F_i(0;a,c)$, first we multiplied the initial number of former smokers in 1993, stratified by age, by the proportions of low, medium and high-intensity ex-smokers arising from the ISTAT EHIS surveys (www.istat.it/it/archivio/167485) carried out in 1994, 1999, 2004, and 2013. Then, we used the distribution of former smokers by time from smoking cessation in 1993 to obtain the initial compartment sizes. This procedure has been applied separately for males and females. For details, see Figures S2.6 and S2.7;
- the relative risks for current and former smokers versus never smokers were obtained from the literature. Specifically, we used the rates estimated from the US population within the Cancer Prevention Study II and reported in the Supplementary Appendix of Thun et al. [4], Tables S4, S11, and S12. For details see Tables S2.1 and S2.2.

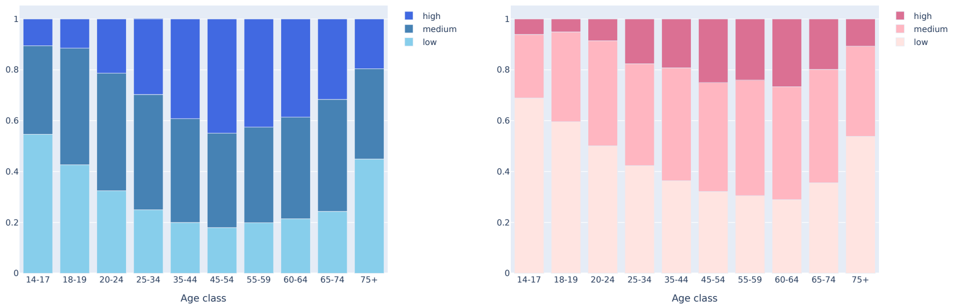

(a) (b)

Fig. S2.1: Average prevalence of smoking intensity among current smokers evaluated over the period 1993-2019 for males (a) and females (b). Source AVQ survey.

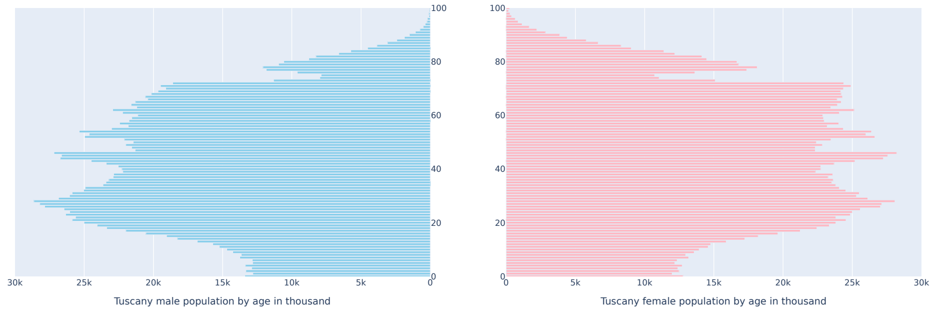

(a) (b)

Fig. S2.2: Size of the population in 1993 for males (a) and females (b). Source ISTAT.

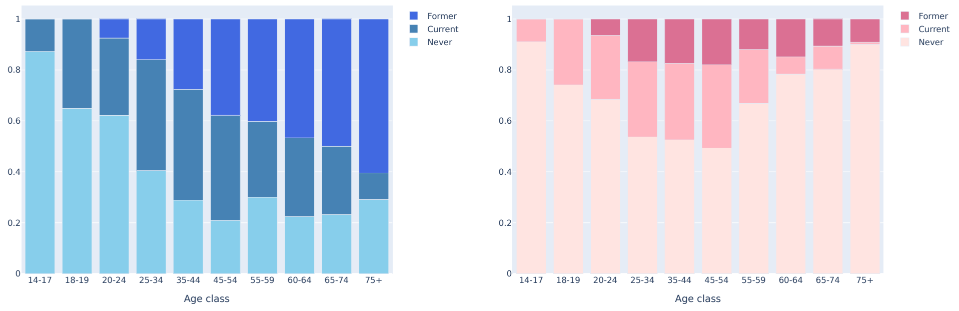

(a) (b)

Fig. S2.3: Prevalence of smoking habits in 1993 for males (a) and females (b). Source AVQ survey.

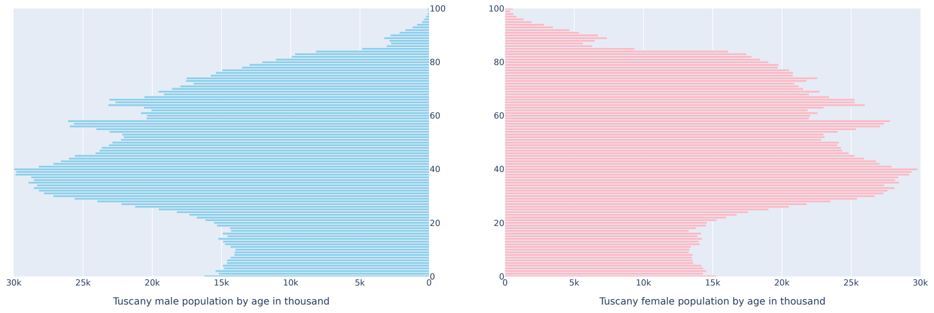

(a) (b)

Fig. S2.4: Size of the population in 2005 for males (a) and females (b). Source ISTAT.

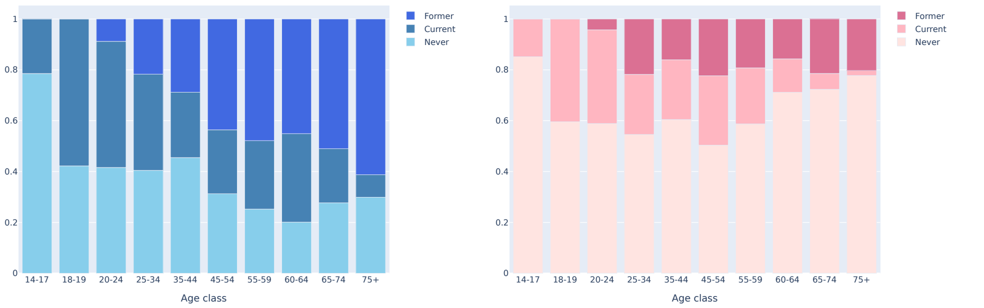

(a) (b)

Fig. S2.5: Prevalence of smoking habits in 2005 for males (a) and females (b). Source AVQ survey.

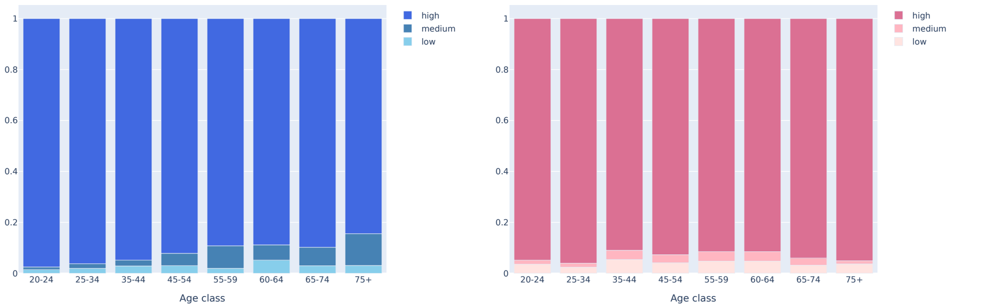

(a) (b)

Fig. S2.6: Average prevalence of smoking intensity among former smokers for males (a) and females (b). Source EHIS survey.

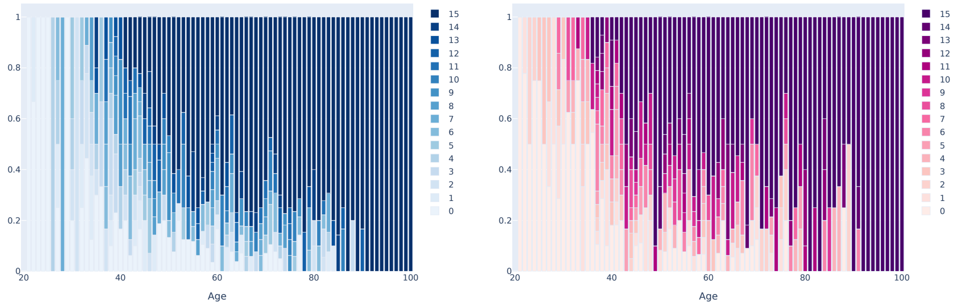

(a) (b)

Fig. S2.7: Average prevalence of time since smoking cessation among former smokers for males (a) and females (b). Source EHIS survey.

Table S2.1: Relative Risk of current smokers by smoking intensity (RR_{C_i}) for males and females. Source Thun et al. [4].

Smoke intensity	Male	Female
**all**	2.43	2.08
**low**	1.91	1.47
**medium**	2.05	1.87
**high**	2.42	2.36

Table S2.2: Relative Risk of former smokers by time since smoking cessation ($RR_{F_i}(c)$) for males and females. Source Thun et al. [4].

Time from cessation	Male	Female
**all**	1.43	1.28
**<2**	2.53	2.26
**2-4**	2.35	2.22
**5-9**	1.90	1.58
**10-19**	1.49	1.29

S3 Additional details on Global Sensitivity Analysis

Table S3.1: Distribution of the input parameters used in the GSA and related data sources.

Parameter	Model	Distribution	Source
$\boldsymbol{\psi} = (\psi_0, \psi_1, \psi_2, \psi_3)$	all	$\psi_i \sim \mathscr{U}(-10, 10)$ independent	non informative distr.
$\boldsymbol{\phi} = (\phi_0, \phi_1, \phi_2, \phi_3)$	all	$\phi_i \sim \mathscr{U}(-10, 10)$ independent	non informative distr.
$\boldsymbol{\omega} = (\omega_0, \omega_1)$	all	$\omega_i \sim \mathscr{U}(0, 10)$ independent	non informative distr.
$\boldsymbol{\pi} = (\pi_{C_l}, \pi_{C_m}, \pi_{C_h})$	males	$\boldsymbol{\pi} \sim \text{Dirichlet}(205^1 \times (0.19, 0.40, 0.41))$	AVQ survey
	females	$\boldsymbol{\pi} \sim \text{Dirichlet}(216^1 \times (0.19, 0.40, 0.41))$	
ν	males	$\nu \sim \text{Poisson}(14,701)$	ISTAT
	females	$\nu \sim \text{Poisson}(13,895)$	
$\boldsymbol{RR}_C = (RR_{C_l}, RR_{C_m}, RR_{C_h})$	males	$\log RR_{C_l} \sim \mathscr{N}_{trun}(\log 1.91, 0.03, 0, \infty)$	Thun et al. [4]
		$\log RR_{C_m} \mid \log RR_{C_l} = a \sim \mathscr{N}_{trun}(\log 2.05, 0.03, a, \infty)$	
		$\log RR_{C_h} \mid \log RR_{C_m} = a \sim \mathscr{N}_{trun}(\log 2.42, 0.02, a, \infty)$	
	females	$\log RR_{C_l} \sim \mathscr{N}_{trun}(\log 1.47, 0.03, 0, \infty)$	
		$\log RR_{C_m} \mid \log RR_{C_l} = a \sim \mathscr{N}_{trun}(\log 1.87, 0.03, a, \infty)$	
		$\log RR_{C_h} \mid \log RR_{C_m} = a \sim \mathscr{N}_{trun}(\log 2.36, 0.03, a, \infty)$	
$RR_F = RR_{F_l}(c) = RR_{F_m}(c) = RR_{F_h}(c) = RR_F(c)$	males	$\log RR_{F_i}(c) \sim \mathscr{N}_{trun}(\log 2.53, 0.03, 0, \infty)$, $c = 1$	Thun et al. [4]
		$\log RR_{F_i}(c) \mid \log RR_{F_i}(1) = a \sim \mathscr{N}_{trun}(\log 2.35, 0.03, 0, a)$, $c \in \{2,3,4\}$	
		$\log RR_{F_i}(c) \mid \log RR_{F_i}(2) = a \sim \mathscr{N}_{trun}(\log 1.90, 0.02, 0, a)$, $c \in \{5,...,9\}$	
		$\log RR_{F_i}(c) \mid \log RR_{F_i}(5) = a \sim \mathscr{N}_{trun}(\log 1.49, 0.02, 0, a)$, $c \geq 10$	
	females	$\log RR_{F_i}(c) \sim \mathscr{N}_{trun}(\log 2.26, 0.05, 0, \infty)$, $c = 1$	Thun et al. [4]
		$\log RR_{F_i}(c) \mid \log RR_{F_i}(1) = a \sim \mathscr{N}_{trun}(\log 2.22, 0.04, 0, a)$, $c \in \{2,3,4\}$	
		$\log RR_{F_i}(c) \mid \log RR_{F_i}(2) = a \sim \mathscr{N}_{trun}(\log 1.58, 0.03, 0, a)$, $c \in \{5,...,9\}$	
		$\log RR_{F_i}(c) \mid \log RR_{F_i}(5) = a \sim \mathscr{N}_{trun}(\log 1.29, 0.03, 0, a)$, $c \geq 10$	
$\boldsymbol{RR}_{status} = (RR_C, RR_F)$	males	$\log RR_C \sim \mathscr{N}_{trun}(\log 2.43, 0.01, 0, \infty)$	Thun et al. [4]
		$\log RR_F \mid \log RR_C = a \sim \mathscr{N}_{trun}(\log 1.43, 0.01, 0, a)$	
	females	$\log RR_C \sim \mathscr{N}_{trun}(\log 2.08, 0.01, 0, \infty)$	Thun et al. [4]
		$\log RR_F \mid \log RR_C = a \sim \mathscr{N}_{trun}(\log 1.28, 0.01, 0, a)$	

[1] Average sample size of the AVQ survey in the period from 1993 to 2019.

S4 Population Attributable Fraction computation

The Population Attributable Fraction for the class of age a at time t, $\text{PAF}(t;a)$, is calculated as the proportion of deaths that would be avoided if all current and former smokers of age a at time t in the population were never smokers [5]:

$$\text{PAF}(t;a) = \frac{\text{SAD}(t;a)}{D_N(t;a) - D_N(t-1;a) + \sum_i (D_{C_i}(t;a) - D_{C_i}(t-1;a)) + \sum_i \sum_c (D_{F_i}(t;a,c) - D_{F_i}(t;a,c))}.$$

Analogously, the overall PAF at time t is:

$$\text{PAF}(t) = \frac{\text{SAD}(t)}{\sum_a ((D_N(t;a) - D_N(t-1;a)) + \sum_a \sum_i (D_{C_i}(t;a) - D_{C_i}(t-1;a)) + \sum_a \sum_i \sum_c (D_{F_i}(t;a,c) - D_{F_i}(t;a,c)))}.$$

S5 Additional results

Table S5.1: Estimated prevalence (%) of never, current, and former smokers in the population with 90% confidence intervals, evaluated every 10 years from 1993 to 2043 for males, by period of calibration.

	**Never**		**Current**		**Former**	
**Year**	**1993 - 2004**	**2005 - 2019**	**1993 - 2004**	**2005 - 2019**	**1993 - 2004**	**2005 - 2019**
**1993**	35.7 (33.6 - 38.0)	-	33.7 (31.7 - 35.5)	-	30.5 (28.2 - 32.9)	-
**2003**	36.9 (35.2 - 38.8)	-	28.7 (28.0 - 30.2)	-	34.4 (32.8 - 35.3)	-
**2013**	40.0 (38.2 - 41.9)	39.2 (36.8 - 41.3)	23.9 (23.2 - 27.2)	25.71 (24.1 - 26.4)	36.1 (33.1 - 37.1)	35.1 (34.0 - 36.7)
**2023**	43.4 (41.2 - 45.8)	43.6 (41.9 - 46.0)	21.1 (20.3 - 25.4)	22.94 (20.8 - 24.2)	35.4 (31.7 - 36.6)	33.5 (31.8 - 35.2)
**2033**	46.7 (43.8 - 49.7)	48.3 (46.8 - 51.1)	19.7 (18.6 - 24.5)	20.95 (18.5 - 22.6)	33.6 (29.5 - 35.0)	30.8 (28.4 - 32.8)
**2043**	49.4 (45.5 - 53.4)	53.1 (51.4 - 56.5)	19.5 (18.0 - 24.1)	19.13 (16.4 - 21.3)	31.1 (27.1 - 33.2)	27.8 (25.2 - 30.0)

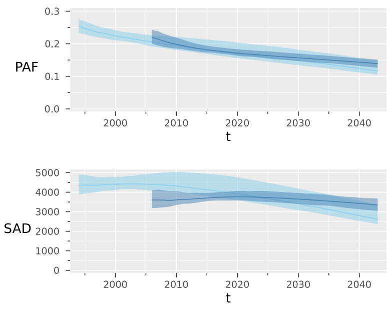

(a)

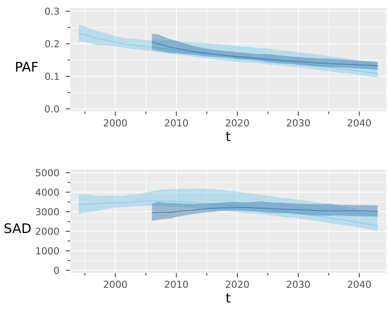

(b)

Fig. S5.1: Results of the two-step estimation procedure for males by period of calibration (from 1993 to 2004 in a light colour and from 2005 to 2019 in a dark colour): Estimated Population Attributable Fraction (PAF) and number of Smoking Attributable Deaths (SAD), with 90% confidence bands, for people over 35 years old (a) and over 65 years old (b).

Table S5.2: Estimated Population Attributable Fraction (PAF) (%) and number of Smoking Attributable Deaths (SAD) in the years 1993, 2003, 2013, 2023, 2033, and 2043, with 90% confidence intervals, among males aged over 35, by period of calibration.

	**PAF**		**SAD**	
**Year**	**1993 - 2004**	**2005 - 2019**	**1993 - 2004**	**2005 - 2019**
**1994**	25.2 (22.9 - 27.9)	-	4,348 (3,829 - 4,979)	-
**2003**	21.3 (19.9 - 22.7)	-	4,376 (4,074 - 4,749)	-
**2013**	18.2 (16.9 - 20.9)	18.7 (17.7 - 22.3)	4,117 (3,776 - 4,778)	3,662 (3,445 - 4,489)
**2023**	15.9 (14.5 - 19.2)	16.7 (16.0 - 22.1)	3,672 (3,341 - 4,501)	3,752 (3,601 - 5,109)
**2033**	13.7 (12.5 - 17.3)	15.2 (14.5 - 21.6)	3,128 (2,875 - 3,975)	3,575 (3,405 - 5,136)
**2043**	11.2 (10.3 - 14.7)	13.7 (12.9 - 19.8)	2,515 (2,305 - 3,331)	3,308 (3,079 - 4,778)

Table S5.3: Estimated Population Attributable Fraction (PAF) (%) and number of Smoking Attributable Deaths (SAD) in the years 1993, 2003, 2013, 2023, 2033, and 2043, with 90% confidence intervals, among males aged over 65, by period of calibration.

	PAF		SAD	
**Year**	**1993 - 2004**	**2005 - 2019**	**1993 - 2004**	**2005 - 2019**
**1994**	23.2 (20.7 - 26.6)	-	3,353 (2,883 - 4,008)	-
**2003**	19.1 (17.8 - 20.9)	-	3,465 (3,166 - 3,768)	-
**2013**	17.0 (15.3 - 19.6)	17.6 (16.4 - 21.3)	3,399 (3,060 - 3,981)	3,100 (2,873 - 3,859)
**2023**	15.0 (13.4 - 18.3)	15.7 (14.9 - 21.4)	3,097 (2,780 - 3,849)	3,207 (3,056 - 4,459)
**2033**	13.0 (11.8 - 16.4)	14.1 (13.5 - 20.8)	2,705 (2,476 - 3,442)	3,059 (2,915 - 4,561)
**2043**	10.4 (9.5 - 13.8)	13.1 (12.3 - 19.4)	2,191 (2,009 - 2,890)	2,994 (2,784 - 4,440)

Table S5.4: Estimated prevalence (%) of never, current, and former smokers in the population with 90% confidence intervals, evaluated every 10 years from 1993 to 2043 for females, by period of calibration.

	**Never**		**Current**		**Former**	
**Year**	**1993 - 2004**	**2005 - 2019**	**1993 - 2004**	**2005 - 2019**	**1993 - 2004**	**2005 - 2019**
**1993**	66.9 (64.8 - 68.5)	-	20.3 (18.9 - 22.3)	-	12.8 (11.5 - 14.4)	-
**2003**	63.5 (61.7 - 64.4)	-	19.7 (19.0 - 20.8)	-	16.8 (15.8 - 17.8)	-
**2013**	60.6 (58.1 - 62.4)	63.5 (61.0 - 65.7)	18.9 (17.5 - 22.1)	17.2 (15.9 - 18.3)	20.5 (16.7 - 22.5)	19.3 (17.9 - 20.9)
**2023**	58.9 (55.7 - 61.9)	63.0 (60.8 - 65.0)	17.9 (16.1 - 22.8)	15.6 (14.5 - 17.6)	23.2 (17.4 - 25.4)	21.4 (19.3 - 22.7)
**2033**	58.9 (55.1 - 63.4)	63.0 (61.0 - 65.3)	16.9 (14.7 - 21.7)	14.1 (12.9 - 17.0)	24.1 (17.2 - 26.5)	22.9 (19.7 - 24.3)
**2043**	60.0 (55.0 - 66.2)	64.4 (62.4 - 67.2)	16.5 (14.0 - 22.4)	12.6 (11.0 - 15.9)	23.5 (16.1 - 26.4)	22.9 (18.8 - 24.4)

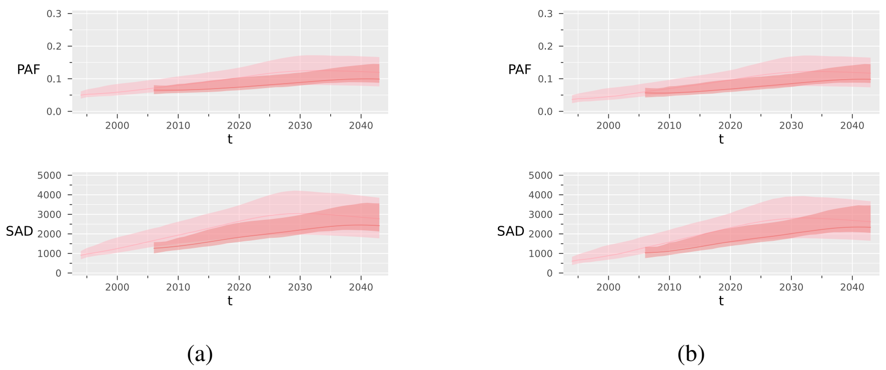

Fig. S5.2: Results of the two-step estimation procedure for females by periods of calibration (from 1993 to 2004 in a light colour and from 2005 to 2019 in a dark colour): Estimated Population Attributable Fraction (PAF) and number of Smoking Attributable Deaths (SAD), with 90% confidence bands, for people over 35 years old (a) and over 65 years old (b).

Table S5.5: Estimated Population Attributable Fraction (PAF) (%) and number of Smoking Attributable Deaths (SAD) in the years 1993, 2003, 2013, 2023, 2033, and 2043, with 90% confidence intervals, among females aged over 35, by period of calibration.

	PAF		SAD	
**Year**	**1993 - 2004**	**2005 - 2019**	**1993 - 2004**	**2005 - 2019**
**1994**	5.0 (4.2 - 6.1)	-	900 (748 - 1110)	-
**2003**	5.8 (5.2 - 8.2)	-	1,301 (1,161 - 1,861)	-
**2013**	7.5 (6.2 - 11.9)	6.5 (5.7 - 9.0)	1,840 (1,544 - 3,020)	1,454 (1,279 - 2,050)
**2023**	9.2 (7.2 - 16.0)	7.7 (6.9 - 10.2)	2,322 (1,809 - 4,115)	1,889 (1,707 - 2,550)
**2033**	9.8 (7.7 - 17.9)	9.1 (8.1 - 12.2)	2,337 (1,841 - 4,364)	2,250 (2,005 - 3,061)
**2043**	8.9 (7.1 - 17.0)	9.5 (8.2 - 13.5)	2,065 (1,656 - 3,937)	2,330 (2,017 - 3,336)

Table S5.6: Estimated Population Attributable Fraction (PAF) (%) and number of Smoking Attributable Deaths (SAD) in the years 1993, 2003, 2013, 2023, 2033, and 2043, with 90% confidence intervals, among females aged over 65, by period of calibration.

	PAF		SAD	
**Year**	**1993 - 2004**	**2005 - 2019**	**1993 - 2004**	**2005 - 2019**
**1994**	3.6 (2.7 - 4.8)	-	605 (448 - 797)	-
**2003**	4.6 (3.9 - 6.9)	-	949 (800 - 1447)	-
**2013**	6.7 (5.6 - 11.0)	5.7 (4.9 - 8.2)	1,554 (1,281 - 2,606)	1,209 (1,030 - 1,785)
**2023**	8.7 (6.6 - 15.3)	7.2 (9.7 - 9.7)	2,063 (1,543 - 3,713)	1,686 (1,501 - 2,303)
**2033**	9.5 (7.4 - 17.8)	8.8 (7.7 - 11.8)	2,167 (1,678 - 4,100)	2,088 (1,826 - 2,851)
**2043**	9.7 (6.7 - 17.0)	9.5 (8.1 - 13.5)	1,938 (1,508 - 3,780)	2,252 (1,917 - 3,223)

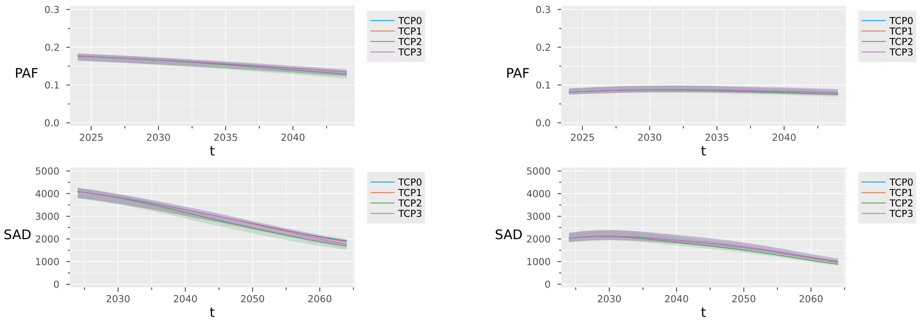

(a) (b)

Fig. S5.3: Estimated Population Attributable Fraction (PAF) and number of Smoking Attributable Deaths (SAD) among people over 35 years of age, with 90% confidence bands, for males (a) and females (b) under different tobacco control policies (TCP).

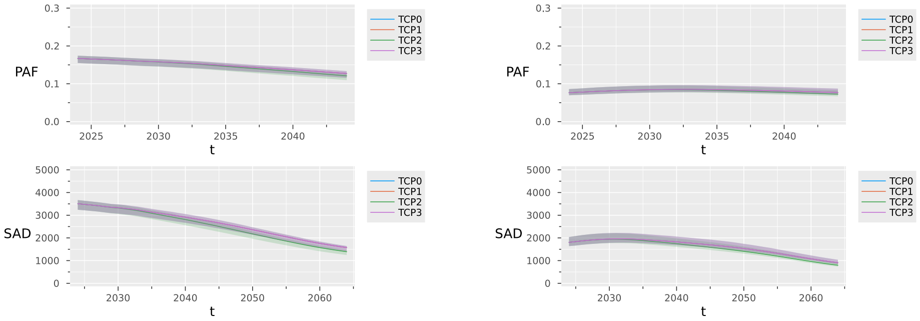

(a) (b)

Fig. S5.4: Estimated Population Attributable Fraction (PAF) and number of Smoking Attributable Deaths (SAD) among people over 65 years of age, with 90% confidence bands, for males (a) and females (b) under different tobacco control policies (TCP).

Table S5.7: Estimated Population Attributable Fraction (PAF) (%) under different tobacco control policies (TCP), in the years 2023, 2033, and 2043, with 90% confidence intervals, among males and females aged over 35 and over 65.

		Male				Female			
**Age**	**Year**	**TCP0**	**TCP1**	**TCP2**	**TCP3**	**TCP0**	**TCP1**	**TCP2**	**TCP3**
**35+**	**2023**	17.6 (16.4 - 18.3)	17.6 (16.4 - 18.3)	17.6 (16.4 - 18.3)	17.6 (16.4 - 18.3)	7.9 (7.0 - 9.6)	7.9 (7.0 - 9.6)	7.9 (7.0 - 9.6)	7.9 (7.0 - 9.6)
	**2033**	15.7 (14.5 - 16.4)	15.7 (14.5 - 16.4)	15.5 (14.3 - 16.4)	15.7 (14.5 - 16.4)	8.6 (7.8 - 10.4)	8.6 (7.8 - 10.4)	8.4 (7.7 - 10.3)	8.6 (7.8 - 10.4)
	**2043**	15.3 (12.1 - 14.0)	13.3 (12.1 - 14.0)	12.6 (11.5 - 14.0)	13.3 (12.1 - 14.0)	7.7 (7.0 - 10.1)	7.7 (7.0 - 10.1)	7.3 (6.6 - 9.7)	7.7 (7.0 - 10.1)
**64+**	**2023**	16.6 (15.5 - 17.4)	16.6 (15.5 - 17.4)	16.6 (15.5 - 17.4)	16.6 (15.5 - 17.4)	7.5 (6.5 - 9.1)	7.5 (6.5 - 9.10)	7.5 (6.5 - 9.1)	7.5 (6.5 - 9.1)
	**2033**	14.9 (13.7 - 15.7)	14.9 (13.7 - 15.7)	14.8 (13.5 - 15.5)	14.9 (13.7 - 15.7)	8.3 (7.6 - 10.2)	8.3 (7.6 - 10.2)	8.2 (7.5 - 10.1)	8.3 (7.6 - 10.2)
	**2043**	12.5 (11.4 - 13.3)	12.5 (11.4 - 13.3)	11.9 (10.8 - 12.7)	11.5 (11.4 - 13.3)	7.5 (6.9 - 9.9)	7.5 (6.9 - 9.9)	7.1 (6.4 - 9.5)	7.5 (6.9 - 9.9)

Table S5.8: Expected percentage decrease in number of Smoking Attributable Deaths (SAD) under different tobacco control policies (TCP1, TCP2, TCP3) with respect to the reference scenario (TCP0), in the years 2023, 2033, and 2043, with 90% confidence intervals, among males and females aged over 35 and over 65.

			Male			Female	
**Age**	**Year**	**TCP1**	**TCP2**	**TCP3**	**TCP1**	**TCP2**	**TCP3**
**35+**	**2033**	-0.0	-1.4	-0.0	-0.0	-1.6	-0.0
	**2043**	-0.1	-5.4	-0.0	-0.1	-6.1	-0.0
	**2053**	-0.5	-9.1	-2.0	-0.3	-9.6	-1.0
	**2063**	-1.8	-11.9	-7.9	-1.3	-12.9	-4.7
**65+**	**2033**	-0.0	-1.4	-0.0	-0.0	-1.6	-0.0
	**2043**	-0.0	-5.4	-0.0	-0.0	-6.1	-0.0
	**2053**	-0.0	-9.1	-0.0	-0.0	-9.5	-0.0
	**2063**	-0.1	-11.7	-0.0	-0.2	-12.8	-0.0

References

[1] Levy, D.T., Gallus, S., Blackman, K., Carreras, G., Vecchia, C.L., Gorini, G.: Italy SimSmoke: the effect of tobacco control policies on smoking prevalence and smoking-attributable deaths in Italy. BMC Public Health **12**(1), 709 (2012) https://doi.org/10.1186/1471-2458-12-709

[2] Hair, E., Bennett, M., Williams, V., Johnson, A., Rath, J., Cantrell, J., Villanti, A., Enders, C., Vallone, D.: Progression to established patterns of cigarette smoking among young adults. Drug and Alcohol Dependence **177**, 77–83 (2017) https://doi.org/10.1016/j.drugalcdep.2017.03.040

[3] Jeong, S.-M., Jeon, K.H., Shin, D.W., Han, K., Kim, D., Park, S.H., Cho, M.H., Lee, C.M., Nam, K.-W., Lee, S.P.: Smoking cessation, but not reduction, reduces cardiovascular disease incidence. European Heart Journal **42**(40), 4141–4153 (2021) https://doi.org/10.1093/eurheartj/ehab578 https://academic.oup.com/eurheartj/article-pdf/42/40/4141/46626087/ehab578.pdf

[4] Thun, M.J., Carter, B.D., Feskanich, D., Freedman, N.D., Prentice, R., Lopez, A.D., Hartge, P., Gapstur, S.M.: 50-Year Trends in Smoking-Related Mortality in the United States. New England Journal of Medicine **368**(4), 351–364 (2013) https://doi.org/10.1056/NEJMsa1211127

[5] GBD 2019 Tobacco Collaborators: Spatial, temporal, and demographic patterns in the prevalence of smoking tobacco use and attributable disease burden in 204 countries and territories, 1990–2019: a systematic analysis from the Global Burden of Disease Study 2019. The Lancet **397**(10292), 2337–2360 (2021) https://doi.org/10.1016/S0140-6736(21)01169-7
